# Evolution of the genetic code: partial optimization of a random code for robustness to translation error in a rugged fitness landscape

**DOI:** 10.1186/1745-6150-2-24

**Published:** 2007-10-23

**Authors:** Artem S Novozhilov, Yuri I Wolf, Eugene V Koonin

**Affiliations:** 1 National Center for Biotechnology Information, National Library of Medicine, National Institutes of Health, Bethesda, MD 20894, USA

## Abstract

**Background:**

The standard genetic code table has a distinctly non-random structure, with similar amino acids often encoded by codons series that differ by a single nucleotide substitution, typically, in the third or the first position of the codon. It has been repeatedly argued that this structure of the code results from selective optimization for robustness to translation errors such that translational misreading has the minimal adverse effect. Indeed, it has been shown in several studies that the standard code is more robust than a substantial majority of random codes. However, it remains unclear how much evolution the standard code underwent, what is the level of optimization, and what is the likely starting point.

**Results:**

We explored possible evolutionary trajectories of the genetic code within a limited domain of the vast space of possible codes. Only those codes were analyzed for robustness to translation error that possess the same block structure and the same degree of degeneracy as the standard code. This choice of a small part of the vast space of possible codes is based on the notion that the block structure of the standard code is a consequence of the structure of the complex between the cognate tRNA and the codon in mRNA where the third base of the codon plays a minimum role as a specificity determinant. Within this part of the fitness landscape, a simple evolutionary algorithm, with elementary evolutionary steps comprising swaps of four-codon or two-codon series, was employed to investigate the optimization of codes for the maximum attainable robustness. The properties of the standard code were compared to the properties of four sets of codes, namely, purely random codes, random codes that are more robust than the standard code, and two sets of codes that resulted from optimization of the first two sets. The comparison of these sets of codes with the standard code and its locally optimized version showed that, on average, optimization of random codes yielded evolutionary trajectories that converged at the same level of robustness to translation errors as the optimization path of the standard code; however, the standard code required considerably fewer steps to reach that level than an average random code. When evolution starts from random codes whose fitness is comparable to that of the standard code, they typically reach much higher level of optimization than the standard code, i.e., the standard code is much closer to its local minimum (fitness peak) than most of the random codes with similar levels of robustness. Thus, the standard genetic code appears to be a point on an evolutionary trajectory from a random point (code) about half the way to the summit of the local peak. The fitness landscape of code evolution appears to be extremely rugged, containing numerous peaks with a broad distribution of heights, and the standard code is relatively unremarkable, being located on the slope of a moderate-height peak.

**Conclusion:**

The standard code appears to be the result of partial optimization of a random code for robustness to errors of translation. The reason the code is not fully optimized could be the trade-off between the beneficial effect of increasing robustness to translation errors and the deleterious effect of codon series reassignment that becomes increasingly severe with growing complexity of the evolving system. Thus, evolution of the code can be represented as a combination of adaptation and frozen accident.

**Reviewers:**

This article was reviewed by David Ardell, Allan Drummond (nominated by Laura Landweber), and Rob Knight.

**Open Peer Review:**

This article was reviewed by David Ardell, Allan Drummond (nominated by Laura Landweber), and Rob Knight.

## Background

**Figure 1:**
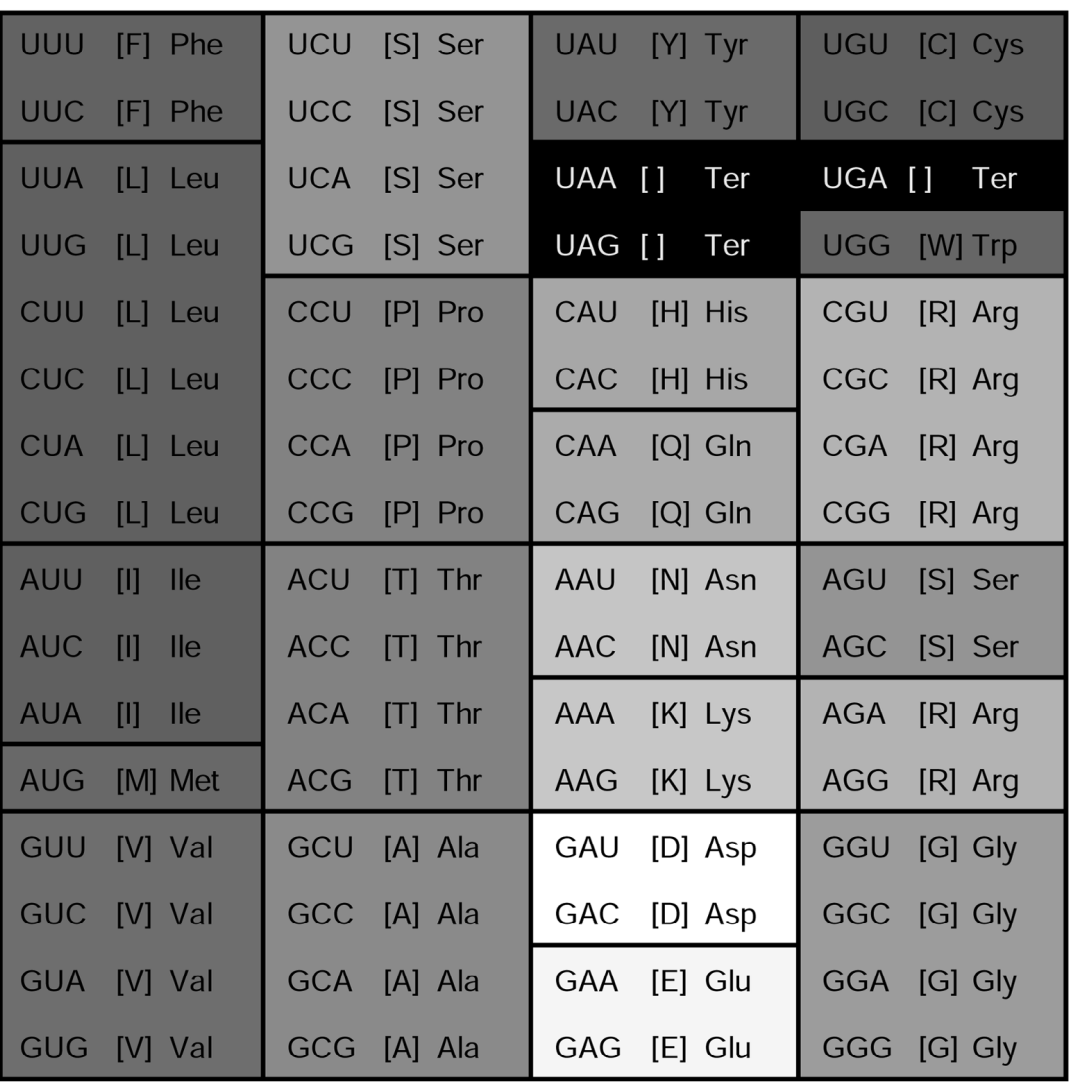
**The standard genetic code**. The codon series are shaded in accordance with the PRS (Polar Requirement Scale) values [6], which is a measure of an amino acid's hydrophobicity: the greater hydrophobicity the darker the shading.

Arguably, one of the most profound, fundamental features of all life forms existing on earth is that, with several minor variations, they share the same genetic code. This standard code is a mapping of 64 codons onto a set of 20 amino acids and the stop signal (Fig. 1). Ever since the standard code was deciphered [1-3], the interplay of the evolutionary forces that shaped the structure of the code has been a subject of debate [3,4]. The main general features that do not depend upon details of the code are: (i) there are four distinct bases in mRNA – two pyrimidines (U, C) and two purines (A, G), (ii) each codon is a triplet of bases, thus forming 64 (4^3^) codons, and (iii) 20 standard protein amino acids are encoded (with the notable exception of selenocysteine and pyrrolysine, for which subsets of organisms have evolved special coding schemes [5]).

The structure of the genetic code is manifestly nonrandom [3]. Given that there are 64 codons for only 20 amino acids, most of the amino acids are encoded by more than one codon, i.e., the standard code is highly redundant; the two exceptions are methionine and tryptophan, each of which is encoded by a single codon. The codon series that code for the same amino acid are, with the single exception of serine, arranged in blocks in the code table and the corresponding codons differ only in the third base position, with the exceptions of arginine and leucine, for which the codon series differ in the first position (Fig. 1). The importance of the nucleotides in the three codon positions dramatically varies: 69% of the point mutations in the third codon position are synonymous, only 4% of the mutations in the first position are synonymous, and none of the point mutations in the second position are synonymous. The structure of the code also, obviously, reflects physicochemical similarities between amino acids; e.g., all codons with a U in the second position code for hydrophobic amino acids (see Fig. 1 where the blocks of synonymous codons are colored with respect to the polar requirement scale [6] (PRS), which is a measure of hydrophobicity). The finer structure of the code comes into view if synonymous codon series that differ by purines or pyrimidines are compared [7]. Related amino acids show a strong tendency to be assigned related codons [3,4,8]. Generally, the standard code is thought to conform with the principles of optimal coding, i.e., the structure of the code appears to be such that it is robust with respect to point mutations, translation errors, and shifts in the reading frame. The block structure of the code is considered to be a necessary condition of this robustness [9].

The fundamental question is how these regularities of the standard genetic code came into being. One of the leading hypotheses is that the primordial code had to reduce errors in translation in order to provide for the efficient synthesis of functional proteins. 'At sufficiently early stages in evolution the fundamental information-transferring processes, i.e., translation, replication, and transcription, must have been error-ridden' [8], so the subsequent evolution is thought to have been driven by selection for an arrangement of the code table that would be increasingly robust to translational misreading – the translation-error hypothesis of code evolution [8,10-15]. The initial evidence for the translation-error hypothesis consists of the aforementioned fact that related codons (codons that differ by one base only), typically, code for related amino acids (amino acids with similar physicochemical properties) and the experimental observations that translational errors occur more frequently in the first and third positions of codons [15-18]. The latter data seem to emphasize the connection between the structure of the code and its robustness to errors of translation as opposed to mutational robustness because, in the latter case, there would be no difference between the effects of mutations in the three positions of the codon. However, Sella and Ardell have argued that minimization of the effect of mutations could be an equally, if not more, important force behind the evolution of the structure of the code than minimization of the effect of translation errors [7,19,20].

Quantitative evidence in support of the translation-error hypothesis has been inferred from comparisons of the standard code with random alternative codes. According to the specified rules (see Results), for each code, a score is calculated, which is used as a measure of the robustness of the code to amino acid replacements induced by translational errors. Often, this score is called "code fitness" [21-23] although it actually represents a measure of "error cost", which is inversely related to "fitness" (i.e., the smaller the score the more robust – or fit – is the respective code); in other instances, the mathematical formulation was transformed such that fitness was calculated directly (the greater the number the fitter the code) [22].

The first Monte Carlo simulation to compare the standard code with random, alternative codes has been described by Alff-Steinberger [11] and indicated that the standard code outperforms most of the random codes if the differential effects of misreading in the first or third base position are taken into account (two sets of 200 codes each were produced). The first reasonably reliable numerical estimates of the fraction of random codes that are more robust than the standard code have been obtained by Haig and Hurst [12] who showed that, if the PRS is employed as the measure of physicochemical similarity of amino acids, the probability of a random code to be fitter than the standard code is *p*_*HH *_≈ 10^-4^. The code error cost score depends on the exact adopted rules such that different cost functions, obviously, have the potential to produce different results. Using a refined cost function that took into account the non-uniformity of codon positions and the assumed transition-transversion bias of translation, Freeland and Hurst [24] have shown that the fraction of random codes that outperforms the standard one is *p*_*FH *_≈ 10^-6^, i.e., "the genetic code is one in a million". Subsequent analyses have yielded even higher estimates of the robustness of the standard code to translation errors [21-23].

Of course, the hypothesis that the code evolved to maximize robustness to errors of translation [14] is by no means the only plausible scenario of the code evolution. The frozen accident hypothesis proposed in Crick's seminal paper [3] posits that, after the primordial genetic code expanded to incorporate all 20 modern amino acids, any change in the code would be lethal, thus ruling out further evolution of the code. The stereochemical hypothesis that can be traced back to the early work of Gamow [3,25-31] postulates that codon assignments for particular amino acids are determined by a physicochemical affinity that exists between the amino acids and the cognate nucleotide triplets (codons or anticodons). Under this hypothesis, the minimization of the effect of translation errors characteristic of the standard code is thought to be an epiphenomenon of purely stereochemical constraints (e.g., similar codons display affinity to amino acids of similar bulk and PRS). This hypothesis implies that the code did not evolve or, in a weak form, that it evolved minimally, adjusting the stereochemical assignments. The stereochemical hypothesis, at least, in its strong form, is readily experimentally testable. However, despite extensive experimentation in this area [32], the reality and relevance of any affinities between amino acid and cognate triplets, codons or anticodons, remain questionable (see [33] for a recent discussion).

The coevolution hypothesis [34-38] posits that the structure of the standard code reflects the biosynthetic pathways of amino acid formation. The coevolution hypothesis agrees with the translation robustness hypothesis in that the genetic code had substantially evolved but differs in defining the main evolutionary process that shaped the standard code table. According to this scenario, the code coevolved with amino acid biosynthesis pathways, i.e., during the evolution of the code, subsets of codons for precursor amino acids have been reassigned to encode product amino acids in the same pathways such that related codons encode metabolically close amino acids. The robustness of the code to translation errors, then, is a byproduct of this coevolutionary process inasmuch as metabolically linked amino acids also tend to be physicochemically similar. However, it has been shown that the coevolution scenario alone does not account for the observed degree of translational error-minimization of the standard code [21,39].

Two major objections to the translation-error hypothesis have been raised [40,41]: (i) although the estimates of *p*_*HH *_and *p*_*FH *_indicate that the standard code is unusual in its robustness to translational errors, the number of alternative codes that are fitter than the standard one is still huge, (ii) the minimization percentage (the relative optimization level reached during genetic code evolution) for the standard code would have been higher (62% for the standard code according to Di Giulio's calculations [42]) if the selection on amino acid distances were the main force. The debate between the translation-error and the coevolution scenarios of the code evolution remains unresolved. The proponents of the translation-error hypothesis reasonably counter the above objections by showing that the distribution of the code error scores (fitness values) has a Gaussian-like shape where the better, more robust codes form a long tail such that the process of adaptation is non-linear, so approaching the absolute minimum is highly improbable [14,30,40,41].

The different hypotheses on code evolution, including the stereochemical hypothesis in its weaker form, are not exclusive. Indeed, as noticed by Knight et al. [30]: "the combination of stereochemical interactions and strong selection could have channeled biosynthetic expansion to produce the current repertoire of 20 coded amino acids". Regardless of the relative contributions of the different evolutionary forces to the organization of the standard code, the high level of the code optimization with respect to errors of translation is in need of an explanation. Here, we describe an analysis of the code fitness landscape using particular error cost functions for codes constructed from blocks that are inherent to the standard genetic code. Evidence is presented that the standard code is a partially optimized version of a random code with the same block structure.

## Results

### The error cost function

The results obtained in this work are closely intertwined with the employed methods; therefore, instead of presenting the latter in a separate Materials and Methods section, we start the Results section with the description of the essential technical details of the analysis and the associated limitations and caveats.

A genetic code is a mapping *a*: *C *→ *A *that assigns an amino acid (or stop signal) *a *∈ *A *to each codon *c *∈ *C*. The error cost function, which is inversely related to code fitness, can be written as

ϕ(a(c))=164∑cf(c)∑c′p(c′|c)d(a(c),a(c′)).
 MathType@MTEF@5@5@+=feaafiart1ev1aaatCvAUfKttLearuWrP9MDH5MBPbIqV92AaeXatLxBI9gBaebbnrfifHhDYfgasaacH8akY=wiFfYdH8Gipec8Eeeu0xXdbba9frFj0=OqFfea0dXdd9vqai=hGuQ8kuc9pgc9s8qqaq=dirpe0xb9q8qiLsFr0=vr0=vr0dc8meaabaqaciaacaGaaeqabaqabeGadaaakeaaiiGacqWFvpGAcqGGOaakcqWGHbqycqGGOaakcqWGJbWycqGGPaqkcqGGPaqkcqGH9aqpdaWcaaqaaiabigdaXaqaaiabiAda2iabisda0aaadaaeqbqaaiabdAgaMjabcIcaOiabdogaJjabcMcaPmaaqafabaGaemiCaaNaeiikaGIafm4yamMbauaacqGG8baFcqWGJbWycqGGPaqkcqWGKbazcqGGOaakcqWGHbqycqGGOaakcqWGJbWycqGGPaqkcqGGSaalcqWGHbqycqGGOaakcuWGJbWygaqbaiabcMcaPiabcMcaPaWcbaGafm4yamMbauaaaeqaniabggHiLdaaleaacqWGJbWyaeqaniabggHiLdGccqGGUaGlaaa@5873@

This formula consists of three main terms. The matrix *p*(*c*'|*c*) is the matrix of relative probabilities that codon *c *is misread as codon *c'*. Considering only codons that differ in one base position, three different matrices *p*(*c*'|*c*) have been considered. Haig and Hurst [12] let *p*(*c*'|*c*) = 1 for any two codons that differ by one base and *p*(*c*'|*c*) = 0 otherwise, not taking into account that transitions (i.e., translational misreading between two purines or two pyrimidines) might occur more often than transversions (interchange between purines and pyrimidines) [43]. Freeland and Hurst [24] additionally introduced a different scheme that incorporates an inferred transition-transversion bias of translational misreading. Under this scheme, *p*(*c*'|*c*) = *tr*_*b *_if *c *and *c' *differ by a transition, and *p*(*c*'|*c*) = 1 if *c *and *c' *differ by a transversion (*tr*_*b *_> 1). Taking into account the different frequencies of translational errors and the different magnitudes of the bias for the three codon positions, Freeland and Hurst [24] proposed the following form for the matrix *p*(*c*'|*c*):

p(c′|c)={1/Nif c and c′ differ in the 3d base only,1/Nif c and c′ differ in the 1st base only and cause a transition,0.5/Nif c and c′ differ in the 1st base only and cause a transversion,0.5/Nif c and c′ differ in the 2nd base only and cause a transition,0.1/Nif c and c′ differ in the 2nd base only and cause a transversion,0otherwise.   
 MathType@MTEF@5@5@+=feaafiart1ev1aaatCvAUfKttLearuWrP9MDH5MBPbIqV92AaeXatLxBI9gBaebbnrfifHhDYfgasaacH8akY=wiFfYdH8Gipec8Eeeu0xXdbba9frFj0=OqFfea0dXdd9vqai=hGuQ8kuc9pgc9s8qqaq=dirpe0xb9q8qiLsFr0=vr0=vr0dc8meaabaqaciaacaGaaeqabaqabeGadaaakeaacqWGWbaCcqGGOaakcuWGJbWygaqbaiabcYha8jabdogaJjabcMcaPiabg2da9maaceaabaqbaeaabyGaaaaabaGaeGymaeJaei4la8IaemOta4eabaGaeeyAaKMaeeOzayMaeeiiaaIaem4yamMaeeiiaaIaeeyyaeMaeeOBa4MaeeizaqMaeeiiaaIafm4yamMbauaacqqGGaaicqqGKbazcqqGPbqAcqqGMbGzcqqGMbGzcqqGLbqzcqqGYbGCcqqGGaaicqqGPbqAcqqGUbGBcqqGGaaicqqG0baDcqqGObaAcqqGLbqzcqqGGaaicqqGZaWmcqqGKbazcqqGGaaicqqGIbGycqqGHbqycqqGZbWCcqqGLbqzcqqGGaaicqqGVbWBcqqGUbGBcqqGSbaBcqqG5bqEcqqGSaalaeaacqaIXaqmcqGGVaWlcqWGobGtaeaacqqGPbqAcqqGMbGzcqqGGaaicqWGJbWycqqGGaaicqqGHbqycqqGUbGBcqqGKbazcqqGGaaicuWGJbWygaqbaiabbccaGiabbsgaKjabbMgaPjabbAgaMjabbAgaMjabbwgaLjabbkhaYjabbccaGiabbMgaPjabb6gaUjabbccaGiabbsha0jabbIgaOjabbwgaLjabbccaGiabbgdaXiabbohaZjabbsha0jabbccaGiabbkgaIjabbggaHjabbohaZjabbwgaLjabbccaGiabb+gaVjabb6gaUjabbYgaSjabbMha5jabbccaGiabbggaHjabb6gaUjabbsgaKjabbccaGiabbogaJjabbggaHjabbwha1jabbohaZjabbwgaLjabbccaGiabbggaHjabbccaGiabbsha0jabbkhaYjabbggaHjabb6gaUjabbohaZjabbMgaPjabbsha0jabbMgaPjabb+gaVjabb6gaUjabbYcaSaqaaiabicdaWiabd6caUiabiwda1iabd+caViabd6eaobqaaiabbMgaPjabbAgaMjabbccaGiabdogaJjabbccaGiabbggaHjabb6gaUjabbsgaKjabbccaGiqbdogaJzaafaGaeeiiaaIaeeizaqMaeeyAaKMaeeOzayMaeeOzayMaeeyzauMaeeOCaiNaeeiiaaIaeeyAaKMaeeOBa4MaeeiiaaIaeeiDaqNaeeiAaGMaeeyzauMaeeiiaaIaeeymaeJaee4CamNaeeiDaqNaeeiiaaIaeeOyaiMaeeyyaeMaee4CamNaeeyzauMaeeiiaaIaee4Ba8MaeeOBa4MaeeiBaWMaeeyEaKNaeeiiaaIaeeyyaeMaeeOBa4MaeeizaqMaeeiiaaIaee4yamMaeeyyaeMaeeyDauNaee4CamNaeeyzauMaeeiiaaIaeeyyaeMaeeiiaaIaeeiDaqNaeeOCaiNaeeyyaeMaeeOBa4Maee4CamNaeeODayNaeeyzauMaeeOCaiNaee4CamNaeeyAaKMaee4Ba8MaeeOBa4MaeeilaWcabaGaeGimaaJaeiOla4IaeGynauJaei4la8IaemOta4eabaGaeeyAaKMaeeOzayMaeeiiaaIaem4yamMaeeiiaaIaeeyyaeMaeeOBa4MaeeizaqMaeeiiaaIafm4yamMbauaacqqGGaaicqqGKbazcqqGPbqAcqqGMbGzcqqGMbGzcqqGLbqzcqqGYbGCcqqGGaaicqqGPbqAcqqGUbGBcqqGGaaicqqG0baDcqqGObaAcqqGLbqzcqqGGaaicqqGYaGmcqqGUbGBcqqGKbazcqqGGaaicqqGIbGycqqGHbqycqqGZbWCcqqGLbqzcqqGGaaicqqGVbWBcqqGUbGBcqqGSbaBcqqG5bqEcqqGGaaicqqGHbqycqqGUbGBcqqGKbazcqqGGaaicqqGJbWycqqGHbqycqqG1bqDcqqGZbWCcqqGLbqzcqqGGaaicqqGHbqycqqGGaaicqqG0baDcqqGYbGCcqqGHbqycqqGUbGBcqqGZbWCcqqGPbqAcqqG0baDcqqGPbqAcqqGVbWBcqqGUbGBcqqGSaalaeaacqqGWaamcqqGUaGlcqqGXaqmcqqGVaWlcqWGobGtaeaacqqGPbqAcqqGMbGzcqqGGaaicqWGJbWycqqGGaaicqqGHbqycqqGUbGBcqqGKbazcqqGGaaicuWGJbWygaqbaiabbccaGiabbsgaKjabbMgaPjabbAgaMjabbAgaMjabbwgaLjabbkhaYjabbccaGiabbMgaPjabb6gaUjabbccaGiabbsha0jabbIgaOjabbwgaLjabbccaGiabbkdaYiabb6gaUjabbsgaKjabbccaGiabbkgaIjabbggaHjabbohaZjabbwgaLjabbccaGiabb+gaVjabb6gaUjabbYgaSjabbMha5jabbccaGiabbggaHjabb6gaUjabbsgaKjabbccaGiabbogaJjabbggaHjabbwha1jabbohaZjabbwgaLjabbccaGiabbggaHjabbccaGiabbsha0jabbkhaYjabbggaHjabb6gaUjabbohaZjabbAha2jabbwgaLjabbkhaYjabbohaZjabbMgaPjabb+gaVjabb6gaUjabbYcaSaqaaiabbcdaWaqaaiabb+gaVjabbsha0jabbIgaOjabbwgaLjabbkhaYjabbEha3jabbMgaPjabbohaZjabbwgaLjabb6caUaaacqqGGaaicqqGGaaicqqGGaaiaiaawUhaaaaa@BCBF@

Here *N *is a normalization constant that is introduced to ensure ∑_*c'*_*p*(*c'*|*c*) = 1 for any *c*. The relative probabilities specified by (2) take into account transition-transversion and translation biases (transitions are twice as frequent as transversions in the first positions and five times as frequent in the second position). The matrix (2) has been used in numerous studies on the evolution of the code [22-24,44,45]. The evidence for differences in the rates of misreading between codon positions comes from experimental analyses ([8,17]; reviewed in [18]). The empirical data on the rate and nature of translational errors indicate that the details that determine the actual error frequencies are quite complex and cannot be adequately described by (2). The only available systematic analysis of translation error rates suggests that the rates of missense errors depend, primarily, on the outcome of the competition between the cognate and non-cognate tRNAs [43]. Nevertheless, qualitatively, the matrix (2) reflects the main pattern in codon misreading during translation such that utilizing (2) instead of simpler alternatives improves the level of optimality of the standard code.

The function *f*(*c*) in (1) quantifies the relative contribution of codon *c *to the overall robustness of a code. In the original studies, this factor was not considered, i.e., *f*(*c*) = 1 was assumed for any *c *[12,24,37,46]. However, Gilis et al. [22] argued that it is natural to expect that a codon misreading event that results in a substitution of a frequent amino acid for another frequent one leads to a greater absolute number of errors and thus has greater consequences for code robustness. Accordingly, Gilis et al. took *f*(*c*) = *fr*_*a*(*c*)_/*n*(*c*), where *fr*_*a*(*c*) _is the relative frequency of amino acid *a*(*c*), and *n*(*c*) is the number of synonymous codons for the amino acid *a*(*c*); taking into consideration the relative amino acid frequencies has been shown to increase the robustness of the standard code effect to translation errors by up to three orders of magnitude (however, see the next section). Another reasonable assumption is that *f*(*c*) represents not amino acid frequencies but the relative codon frequencies, and so, codon usage differences among taxa are incorporated [45] such that *f*(*c*) = *fr*_*c*_. Alternatively, the codon usage differences were considered under the assumption that the GC content of primordial genomes was much higher than in extant genomes, so random frequencies were assigned to codons proportionally to their GC content [44]. Both studies have shown that incorporation of codon usage differences reduces the robustness of the standard code.

The distance *d*(*a*(*c*), a(*c'*)) in (1) defines the cost of replacing amino acid *a*(*c*) with amino acid *a*(*c'*). We consider several possible choices for this function. In many cases, the PRS is used in the form *d*(*a*(*c*), a(*c'*)) = *h*(*a*(*c*) - *h*(*a*(*c'*)))^2^, where *h*(*a*) is the value for the amino acid *a *in PRS. Gilis et al. [22] proposed the function *d*(*a*(*c*), a(*c'*)) that has been inspired by computations of the change in the free energy of a protein resulting from a given amino acid substitution; we denote this cost measure the Gilis scoring matrix. Another prominent choice of the distance function is PAM74-100 [47]. This matrix has been first proposed for quantification of code robustness by Ardell [46]. Freeland et al. [21] discuss potential difficulties arising from the fact that this empirically derived substitution matrix might reflect the standard code itself, and only indirectly, the differences between amino acids. They suggest that PAM74-100 is the only potentially acceptable choice amongst the PAM series of matrices because this matrix was obtained from alignments of highly diverged protein sequences unlike other PAM matrices that were derived from close homologs and manipulated mathematically to derive substitution patterns at high divergence (see also [48]).

In this work, to compare the effect of different matrices on the code optimization, we used the PRS, the Gilis scoring matrix, PAM74-100, PAM250, and BLOSUM80 (see the next section). Prior to the actual computations, all the matrices were transformed to the form *d*_*ij *_= (*D*_*ii *_+ *D*_*jj *_- 2D_*ij*_)/2, where *D*_*ij *_is the original similarity score for a pair of amino acids, such that the resulting matrix is symmetric with zeroes on the main diagonal. Hence larger values of the function (1) indicate a lower robustness of a code; *d*(*a*(*c*), a(*c'*)) is taken equal to zero if *a*(*c*) or *a*(*c*') is a stop codon.

### Random codes

The total number of possible genetic codes, assuming that each of the 20 amino acids and the stop signal have to be coded by at least one codon, is vast: *N*_*T *_≈ 1.51·10^84^. Therefore, to generate random codes, a subset of the possible codes is usually selected by different criteria. The algorithm commonly used to generate random codes keeps the block structure of the standard code intact, i.e., amino acids are assigned to four-codon series and two-codon series (and two single codons) positioned as they are in the standard code table, and the stop codons are fixed at their positions as well (Fig. 1). The premise behind this choice is that the block structure of the code is a direct, mechanistic consequence of the mode of interaction between the ribosome, mRNA, and the cognate tRNA. Indeed, structural analyses of the ribosome co-crystallized with cognate tRNAs have shown that the ribosome recognizes the detailed geometry of the first two base pairs of the codon but is less stringent with respect to the third position [49-51], providing a mechanistic basis for Crick's wobble hypothesis [2]. Accordingly, organization of codon series identical or close to that in the standard code is likely to have been an inherent feature of the code that was in place already in the earliest functional translation system or, at least, at a very early stage of the code's evolution. This is not to be taken to mean that codes with different block structures are unviable or "impossible" but they are likely to be substantially less fit than those with the canonical block structure. Because, in practical terms, the entire code space is intractable and restrictions are required for any simulations to be completed, the choice of codes with the standard block structure appears best justified. The amino acids, then, are shuffled between these blocks such that there are 20! ≈ 2.4·10^18 ^possible alternative codes. In most of the previous studies on the robustness of the standard code [12,22-24,44,45], this algorithm was used to generate random alternative codes, sometimes, with modifications to prohibit particular permutations; however, analyses of random code generated with more general algorithms, without the constraint on the block structure, also have been reported [13,52,53].

One feature of the above algorithm is that it changes the number of synonymous codons coding for a particular amino acid, e.g., in most realizations, methionine and tryptophan, the two amino acids that are encoded by a single codon in the standard code, are assigned to codon series consisting of 2, 4 or 6 synonymous codons. A negative correlation has been shown to exist between the size (molecular weight) of an amino acid and the number of codons allocated to it, i.e., small amino acids typically have four-codon series whereas larger amino acids tend to have two-codon series [29,54]. Another feature of the standard code is the strong, positive correlation between the number of synonymous codons and amino acid frequencies in proteins. Although these frequencies differ from protein to protein and from species to species, there is a clear, prevailing trend of abundant amino acids to possess larger codon series than rare amino acids (e.g., such abundant amino acids as Leu and Ser are each encoded by 6 codons [22,55]). Therefore, it appears reasonable to consider additionally constrained random codes that retain the degeneracy pattern of codons inherent to the standard code (i.e., each amino acid is allotted the same number of codons as in the standard code). Accordingly, we implemented the following procedure that generates ≈ 10^19 ^random codes:

1. The stop codons and Trp are kept at their positions in the standard code table (Fig. 1);

2. The assignments of the 8 amino acids (Ser, Leu, Pro, Arg, Thr, Val, Ala, Gly) that are encoded by four-codon series are distributed randomly among the 14 blocks of the code table. A block is defined as four codons that differ only in the third position irrespective of whether they encode one or two amino acids. There are, obviously, 16 blocks altogether; the two blocks that contain stop codons are excluded from the randomization procedure.

3. The assignments of the remaining amino acids (Phe, Leu, Tyr, Cys, His, Gln, Ile, Ile+Met [operationally treated as one amino acid], Asn, Lys, Ser, Arg, Asp, Glu) that are encoded by two-codon series are distributed randomly among the remaining 14 empty two-codon half-blocks (there are 16 unoccupied half-blocks altogether but two half-blocks containing stop codons were excluded from the randomization procedure).

**Figure 2:**
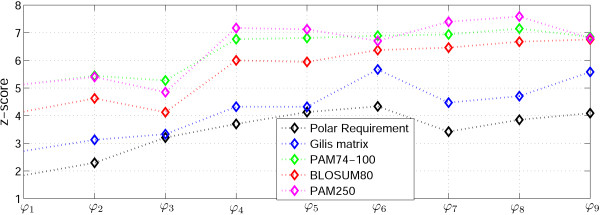
**Comparison of the standard code with random alternatives for different amino acid substitution matrices and cost functions (1)**. Z-score is the distance, measured in standard deviations, between the mean of random code costs and the standard code cost. *ϕ*_1_, *ϕ*_2_, *ϕ*_3 _are the cost functions (1) where *f*(*c*) is the frequency of codon *c*; *ϕ*_4_, *ϕ*_5_, *ϕ*_6 _are the cost functions (1) for *f*(*c*) = 1 *ϕ*_7_, *ϕ*_8_, *ϕ*_9_;are the cost functions (1) where *f*(*c*) is the respective amino acid frequency; in *ϕ*_1_, *ϕ*_4_, *ϕ*_7 _*p*(*c*'|*c*) = 1 for any *c *and *c*' that differ by 1 nucleotide, and *p*(*c*'|*c*) = 0 otherwise; *ϕ*_2_, *ϕ*_5_, *ϕ*_8 _incorporate the inferred transition-transversion bias, i.e., *p*(*c*'|*c*) = *tr*_*b *_if *c *and *c*' differ by a transition, and *p*(*c*'|*c*) = 1 if *c*and *c*' differ by a transversion (*tr*_*b *_= 2 in our calculations); *ϕ*_3_, *ϕ*_6_, *ϕ*_9 _use the scheme (2).

We then estimated the fraction of random codes that outperform the standard code using different error cost functions described in the preceding section. Briefly, (i) codon frequencies typical of extant genomes seem to be suboptimal with respect to the code robustness (error cost) because taking them into account in (1) decreases the difference between the standard code and the random codes (see Fig. 2, cost functions *ϕ*_1_, *ϕ *_2_, *ϕ *_3_); similar observations have been reported previously [44,45,56]; (ii) for the PRS and the Gilis matrix cost measures, the inclusion of the inferred translation bias [i.e., the differences in misreading frequencies between the nucleotide positions in a codon together with the positional transition-transversion bias as represented in (2)], improves the code robustness; by contrast, for the PAM and BLOSUM matrices, the scheme (2), usually, has no significant effect or even reduces the code robustness when compared to calculations that take into account only the transition-transversion bias [e.g., cost functions *ϕ*_5_, *ϕ *_6_, where *ϕ*_5 _incorporates transition-transversion bias only, whereas *ϕ*_6 _is the cost function using (2), see also pairs of cost functions *ϕ*_2_, *ϕ *_3 _and *ϕ*_8_, *ϕ *_9 _(Fig. 2)); (iii) the PAM and BLOSUM matrices show a higher level of robustness for the standard code when compared to the PRS and the Gilis score matrix; (iv) the PAM 74-100 matrix showed results very similar to those obtained with PAM 250 or BLOSUM 80, suggesting that it is equally inadequate as a measure for the cost of amino acid substitutions, as previously proposed by others [48,57]; (v) inclusion of amino acid frequencies, according to [22], had no effect on the genetic code optimality, in contrast to the results of Gilis et al. [22], this can be attributed to the fact that, in our algorithm, each amino acid is coded for by the same number of codons as in the standard code [compare the cost functions *ϕ*_7_, *ϕ *_8_, *ϕ*_9_, in which amino acid frequencies are included, with *ϕ*_4_, *ϕ *_5_, *ϕ*_6_, where no differential weights are assigned to codons (Fig. 2)].

Having assessed the results of the comparisons of the standard code with simulated random codes, we decided to use (1) with *f*(*c*) = 1, the relative probability matrix (2) because these forms of the cost function yielded the maximum differentiation of the standard code from random codes (this cost functions is designated *ϕ*_6_, in Fig. 2). Specifically, the fraction of the codes that outperform the standard one is ≈ 4·10^-6 ^for PRS and ≈ 1.6·10^-6 ^for the Gilis matrix. These two amino acid substitution cost matrices were the only ones employed for further analysis. The other examined matrices, the PAMs and the BLOSUMs, were deemed inapplicable for modeling the code evolution, essentially, because these matrices have been derived from comparisons of protein sequences that are encoded by the standard code, and so cannot be independent of that code; as shown above, this applies to PAM74-100 as well as to the classic PAM matrices [48].

### Searching the fitness landscape

To perform a local search of the space of possible genetic codes we implement a simple, greedy minimization algorithm:

1. All possible pairwise swaps of amino acid assignments among the 14 of the 16 four-codon blocks (except for the two blocks that contain stop codons; Fig. 1) are considered (91 swaps altogether); the swap that yields the greatest improvement of the code is chosen.

2. Repeat step 1 with the new code table until there is no improvement.

3. The 8 four-codon series that encode one amino acid each (see above) are excluded from the search, and all possible swaps of the amino acid assignments among the remaining 14 two-codon half-blocks are considered (another 91 swaps), with the two half-blocks containing stop codons excluded from the search. The swap that yields the greatest improvement is chosen.

4. Repeat step 3 with the new code table until there is no improvement.

5. Steps 1–4 are repeated until there is no improvement.

Using this fully deterministic algorithm we can find the shortest evolutionary trajectory from a given starting code to its local minimum of the error cost function (i.e., to a local fitness peak), under the assumption that each step involves the greatest locally available decrease of the error cost. Each evolutionary step is a pairwise swap of codon quadruplets that differ in the first or second position (or both) or swap of codon pairs that may differ in any of the three codon positions. The codes that are produced by this algorithm have the same structure as is inherent to the algorithm producing random codes.

### The maximum-fitness amino acid substitution matrix for the given code

It is instructive to analyze the relationships between the genetic code and its fitness from a reverse perspective, i.e., to derive the best matrix of amino acid substitution costs for a given (e.g., standard) code. A comparison of such hypothetical, optimal score matrix with known matrices would highlight the sources of "sub-optimality" of the standard code.

Formally, let us consider the following problem:

min⁡d(a(c),a(c′))ϕ(d)
 MathType@MTEF@5@5@+=feaafiart1ev1aaatCvAUfKttLearuWrP9MDH5MBPbIqV92AaeXatLxBI9gBaebbnrfifHhDYfgasaacH8akY=wiFfYdH8Gipec8Eeeu0xXdbba9frFj0=OqFfea0dXdd9vqai=hGuQ8kuc9pgc9s8qqaq=dirpe0xb9q8qiLsFr0=vr0=vr0dc8meaabaqaciaacaGaaeqabaqabeGadaaakeaadaWfqaqaaiGbc2gaTjabcMgaPjabc6gaUbWcbaGaemizaqMaeiikaGIaemyyaeMaeiikaGIaem4yamMaeiykaKIaeiilaWIaemyyaeMaeiikaGIafm4yamMbauaacqGGPaqkcqGGPaqkaeqaaGGacOGae8x1dOMaeiikaGIaemizaqMaeiykaKcaaa@426E@

for the standard genetic code *a*(*c*), *ϕ *(*d*) is given by (1). The function *ϕ *in (1) is linear with respect to unknown *d*_*ij*_, so the trivial solution is *d *≡ 0. To impose reasonable constraints on *d*_*ij*_, we used either the matrix *d*^*P*^, obtained from the PRS, or the Gilis matrix *d*^*G*^. The choice of possible constraints is broad and wide open; we decided to set the average cost of the substitution of any other amino acid for amino acid *i *to be equal to the average cost from *d*^*P *^or *d*^*G*^; in addition, it was required that the cost of substitution of any other amino acid for amino acid *i *was equal to or less than the substitution maximum cost for amino acid *i *in *d*^*P *^or *d*^*G*^:

∑jdij=∑jdijC for any i=1,...,20,dij≤max⁡jdijC for any i,j=1,...,20.
 MathType@MTEF@5@5@+=feaafiart1ev1aaatCvAUfKttLearuWrP9MDH5MBPbIqV92AaeXatLxBI9gBaebbnrfifHhDYfgasaacH8akY=wiFfYdH8Gipec8Eeeu0xXdbba9frFj0=OqFfea0dXdd9vqai=hGuQ8kuc9pgc9s8qqaq=dirpe0xb9q8qiLsFr0=vr0=vr0dc8meaabaqaciaacaGaaeqabaqabeGadaaakeaafaqaaeGabaaabaWaaabuaeaacqWGKbazdaWgaaWcbaGaemyAaKMaemOAaOgabeaaaeaacqWGQbGAaeqaniabggHiLdGccqGH9aqpdaaeqbqaaiabdsgaKnaaDaaaleaacqWGPbqAcqWGQbGAaeaacqWGdbWqaaaabaGaemOAaOgabeqdcqGHris5aOGaeeiiaaIaeeOzayMaee4Ba8MaeeOCaiNaeeiiaaIaeeyyaeMaeeOBa4MaeeyEaKNaeeiiaaIaemyAaKMaeyypa0JaeGymaeJaeiilaWIaeiOla4IaeiOla4IaeiOla4IaeiilaWIaeGOmaiJaeGimaaJaeiilaWcabaGaemizaq2aaSbaaSqaaiabdMgaPjabdQgaQbqabaGccqGHKjYOdaWfqaqaaiGbc2gaTjabcggaHjabcIha4bWcbaGaemOAaOgabeaakiabdsgaKnaaDaaaleaacqWGPbqAcqWGQbGAaeaacqWGdbWqaaGccqqGGaaicqqGMbGzcqqGVbWBcqqGYbGCcqqGGaaicqqGHbqycqqGUbGBcqqG5bqEcqqGGaaicqWGPbqAcqGGSaalcqWGQbGAcqGH9aqpcqaIXaqmcqGGSaalcqGGUaGlcqGGUaGlcqGGUaGlcqGGSaalcqaIYaGmcqaIWaamcqGGUaGlaaaaaa@7BB4@

Here the superscript *C *stands for *P *(polar requirement) or *G *(the Gilis matrix). Additional constraints are given by *d*_*ij *_≥ 0, *d*_*ij *_= *d*_*ji*_, *d*_*ii *_= 0, so there is a well-posed linear programming problem for each cost measure.

Although the matrix *d*, in general, has 190 different elements, in (3) only 75 of these are included because the error cost function (1) takes into account only pairs of amino acids whose codons differ by one nucleotide. A random code will almost always have a different set of single-substitution elements than the standard code. Therefore, although the matrix *d *obtained by solving (3) for the standard code does not guarantee that the standard code becomes ***the ***most robust one, it is reasonable to expect that, in general, the optimization level of the standard code with this new matrix will be higher than with the matrix that was used to write down the constraints for the optimization problem.

In Table 1, we compare the performance of the standard code when using simultaneously a given cost measure (PRS or the Gilis matrix) and the solution of (3) obtained with the constraints inferred for this cost measure. As expected, substitution of the solutions of (3) into (1) makes the standard code more robust to translational errors than substitution of the PRS or the Gilis matrix. Whereas the new, optimized matrix *d *reduces the error cost (increases the fitness) of the standard code, the mean of the random code cost distribution remains the same, so the distance from the standard code to this mean increases when measured in standard deviation units (Table 1). The PRS proves to be more robust to the matrix optimization procedure as the correlation between the solution of (3) and the original matrix was much greater (*R *= 0.92) than for the Gilis matrix (*R *= 0.67).

**Table 1:** Comparison of the standard code with random alternatives

**Cost measure**	*ϕ*	*μ*	*σ*	*z*-score	*R*
*PRS*	2.65	6.87	0.97	4.34	0.92
*Optimized solution*	2.11	6.85	0.98	4.83	
*Gilis matrix*	4.01	4.68	0.12	5.67	0.60
*Optimized solution*	2.9	4.63	0.2	8.59	

*Note: *The solutions of problem (3) compared with the cost measures (PRS and Gillis matrix) from which the constraints were inferred. *ϕ *is the error cost value of the standard code, *μ *is the mean of random code costs, *σ *is the standard deviation for the distribution of random code cost values, *z*-score is the distance from *ϕ *to *μ *in standard deviations: z = (*μ *- *ϕ*)/*σ*; *R *is the correlation coefficient between those entries of the cost function that are present in (3)

Using the solutions of (3), it is possible to identify those pairs of amino acids for which the cost of replacement in the PRS or the Gilis matrix is too high, leading to the suboptimality of these amino acid substitution matrices vis-a-vis the standard code. Consider, e.g., the matrix *d*^*P *^obtained from the PRS and the solution of (3) *d *with the corresponding constraints. Examination of the difference Δ = *d *- *d*^*P *^allows one to identify those pairs of amino acids *i *and *j *for which the cost of replacement should be decreased at the expense of other amino acids, in order to optimize the matrix; these are the negative elements in Δ (Table 2). The list of these "suboptimal" amino acid pairs is distinctly non-random. Most of the amino acids in these pairs come from the 4^th ^(middle G) and, to a lesser extent, the third (middle A) columns of the standard codon table (Fig. 1). Indeed, the juxtaposition of arginine with tryptophane, glycine, cysteine and serine in the 4th column of the standard code is an obvious source of the most costly translation errors, given the major differences in the physico-chemical properties of these amino acids (which translated into high costs in empirically derived substitution matrices, such as PAMs and BLOSUMs). Intuitively, if one were given the task of improving the code, it would seem a natural idea to start with changing the position of arginine such that it is not linked to the above amino acids through single nucleotide substitutions. Indeed, the suboptimal encoding of arginine in the standard code has been noticed previously, leading to the suggestion that arginine was a late addition to the code [58-61].

**Table 2:** The pairs of amino acids that show the highest negative impact on the standard code fitness

PRS			Gilis score matrix			Average		
Arg	Cys	-1.00	Arg	Gly	-1.00	Arg	Trp	-0.70
Arg	Trp	-0.82	Leu	Phe	-1.00	His	Tyr	-0.60
His	Leu	-0.66	Ala	Thr	-0.93	Arg	Gly	-0.54
Asn	Tyr	-0.52	Arg	Ser	-0.71	Leu	Phe	-0.50
His	Tyr	-0.49	Asn	Lys	-0.71	Arg	Cys	-0.50
Glu	Lys	-0.31	Asp	Glu	-0.71	Ala	Thr	-0.47
Lys	Thr	-0.18	Gln	His	-0.71	Arg	Ser	-0.43
Leu	Pro	-0.16	His	Tyr	-0.71	Glu	Lys	-0.41
Arg	Ser	-0.14	Ile	Met	-0.57	Asp	Glu	-0.36
Phe	Ser	-0.13	Arg	Trp	-0.57	Gln	His	-0.36
Gln	Lys	-0.12	Glu	Lys	-0.50	Asn	Lys	-0.36
Ala	Val	-0.11	Asn	Asp	-0.43	His	Leu	-0.33
Lys	Met	-0.11	Leu	Val	-0.43	Ile	Met	-0.29
Gly	Trp	-0.09	Ala	Ser	-0.36	Leu	Val	-0.23
Met	Thr	-0.09	Pro	Ser	-0.29	Asn	Tyr	-0.19

*Note: *The negative elements were normalized such that the smallest one equals -1; the 15 most significant pairs are shown.

### Modeling the evolution of the code along the path of increasing robustness to errors of translation

In the previous section we identified the pairs of amino acids whose placement in the standard code table is the worst with respect to the error cost. These findings suggest that the standard code and, obviously, the huge majority of random codes can be improved by a series of simple amino acid swaps. However, the actual sequence of swapping steps that would lead to increased code fitness remained to be determined. To this end, we developed the simple, greedy minimization algorithm described above.

Using this minimization algorithm and the algorithm for producing random codes, we generated four sets of genetic codes for each of the two error cost functions: **r**, random codes (300 random codes were generated); **o**, the codes obtained from **r **as a result of minimization; **R**, random codes that outperform the standard code (100 codes were found as a result of random search); **O**, the codes obtained from **R **as a result of minimization. We then compared the standard code with each of these four sets in an attempt to determine whether or not the standard code is likely to be a result of evolutionary optimization for translation robustness.

**Figure 3:**
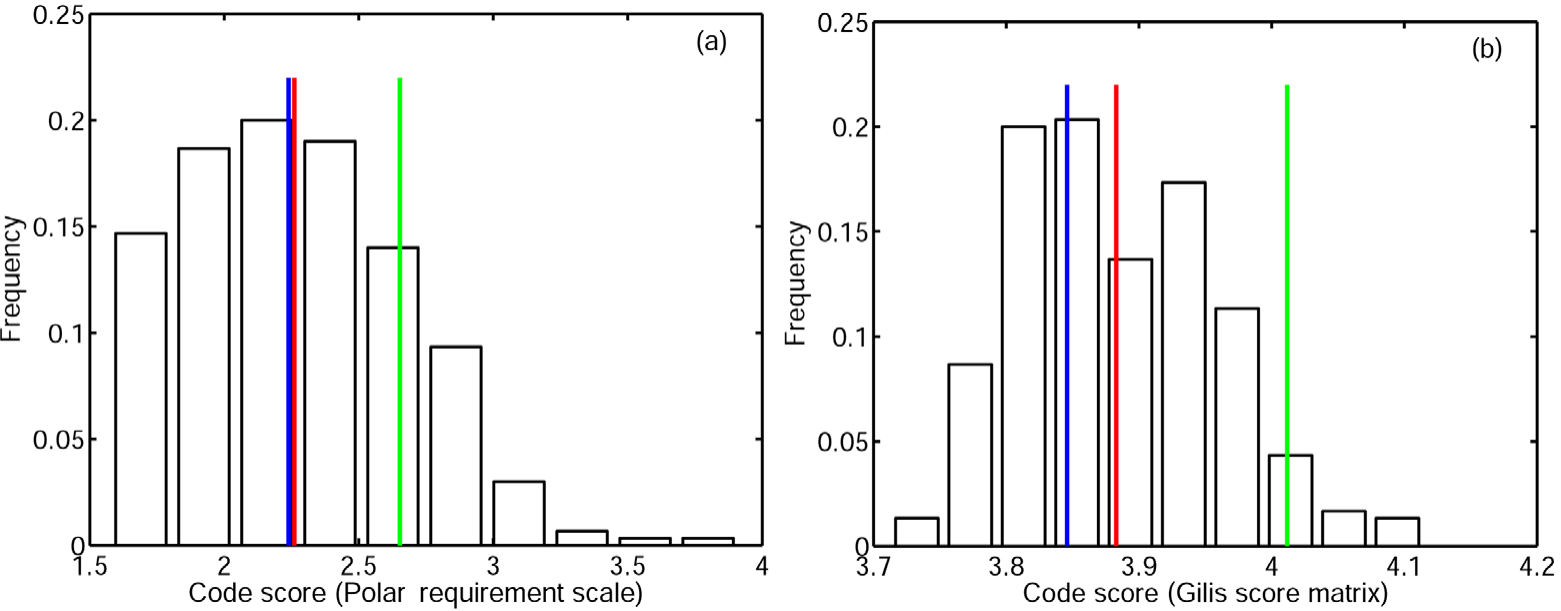
**Distribution of code scores (set **o**) obtained as a result of optimization of random codes**. The green line is the cost of the standard code, the blue line is the cost of the code which was obtained by minimization of the standard code, the red line is the mean of the distribution. (a)PRS; (b)Gilis matrix.

When the error cost of random codes was minimized by using the straightforward procedure described that includes only pairwise swaps at each step, 81% of the random codes for the PRS and 94% of the random codes for the Gilis matrix reached error cost values that were lower than the cost of the standard code (Fig. 3). More strikingly, 50% of the random codes for the PRS and 40% of the random codes for the Gilis matrix could be minimized to cost values that were lower than the cost of the code reached upon minimization of the standard code (Fig. 3).

Thus, in spite of the fact that we start with random codes (within the restrictions of the block structure of the code table imposed by the randomization algorithm), the simple minimization procedure we employ, in most cases, leads to codes that have a greater fitness than the standard code. Moreover, approximately half of the random codes reach fitness levels that are greater than the maximum fitness attainable when the error cost of the standard code is minimized using the same procedure. Thus, although the standard code is much more robust to translation errors than an average random code, the employed minimization procedure brings the standard code to about the same level of optimality as a randomly picked code; of course, for the latter, to reach that minimal level, takes more swapping steps. These observations suggest that the code fitness landscape is highly rugged, i.e., consists of numerous fitness peaks of, approximately, the same height.

**Figure 4:**
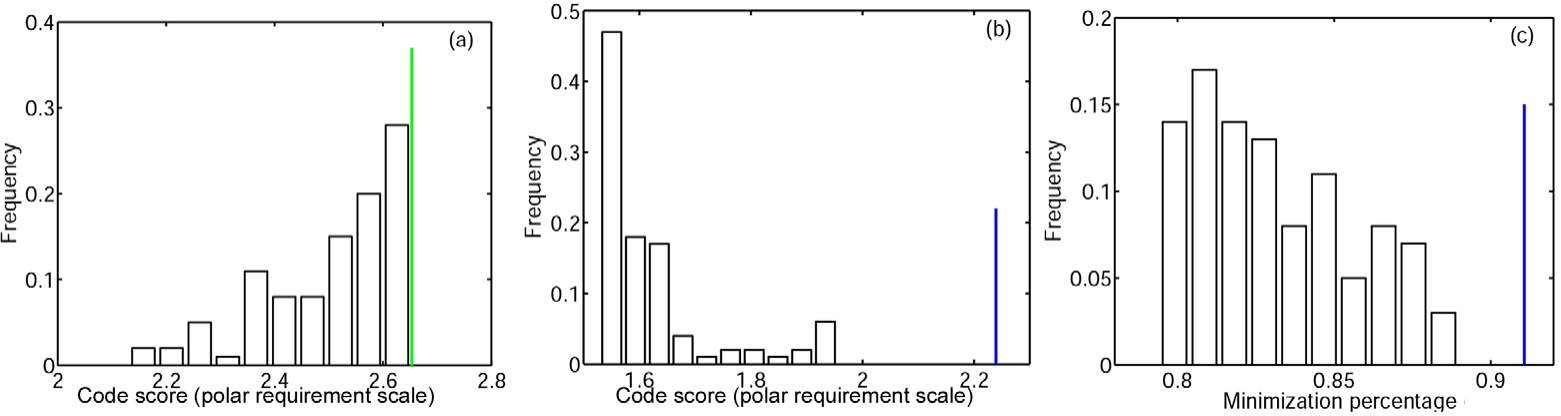
**The results of optimization of the random codes with cost values lower than the cost of the standard code (set **R**)**. The PRS was used as the measure of amino acid substitution cost. (a) Distribution of the code scores from **R**; the green line is the cost of the standard code; (b) Distribution of the scores for the codes obtained by optimization of the codes from **R **(set **O**), the blue line is the cost of the code obtained by optimization of the standard code; (c) Minimization percentage of the codes from **O **(see text for details); the blue line is the minimization percentage of the standard code.

**Figure 5:**
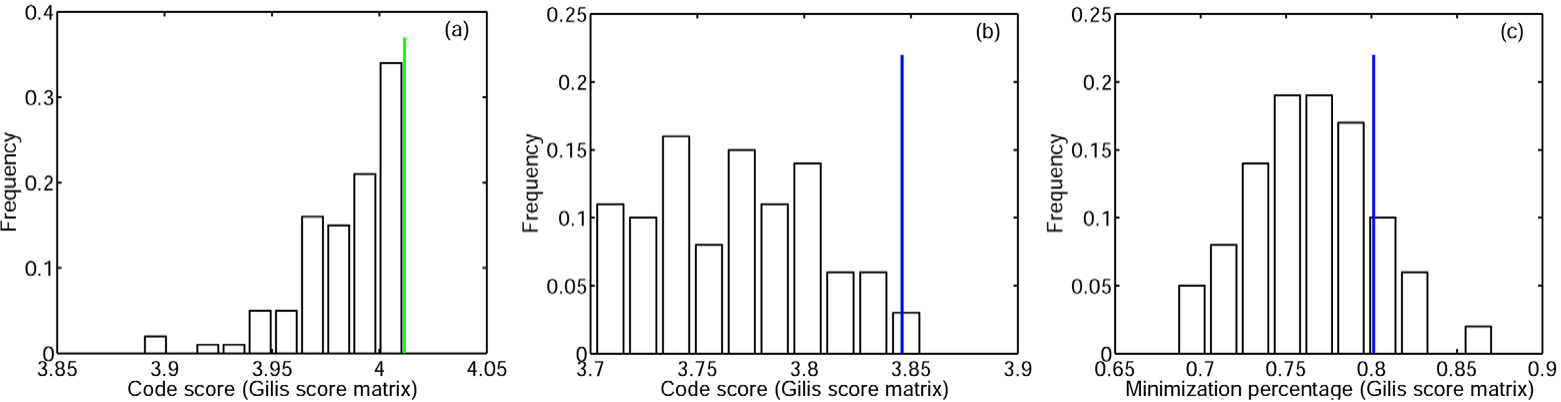
**The results of optimization of the random codes with cost values lower than the cost of the standard code (set **R**)**. The Gillis matrix was used as the measure of amino acid substitution cost. (a) Distribution of the code scores from **R**; the green line is the cost of the standard code; (b) Distribution of the scores for the codes obtained by optimization of the codes from **R **(set **O**), the blue line is the cost of the code obtained by optimization of the standard code; (c) Minimization percentage of the codes from **O **(see text for details); the blue line is the minimization percentage of the standard code.

We then explored the results of minimization of the subset of the random codes, **R**, that outperform the standard code (Figs. 4, 5). For each of the codes from **R**, we also computed the minimization percentage, i.e., the degree of optimization that is attainable for the given code along its specific evolutionary trajectory on the fitness landscape. For example, for the standard code, if *ϕ*_*st *_is the error cost, *ϕ*_min _is the error cost of the best code that was obtained from the standard code using the minimization algorithm, and *μ *is the mean of the distribution of random code scores, than minimization percentage *MP *is given by *MP *= (*μ *- *ϕ*_*st*_)/(*μ *- *ϕ*_min_). The minimization percentage of the standard code is 91% for the PRS and 80% for the Gilis matrix.

For almost all codes from **R**, the minimization procedure led to codes (**O**) that were better than the optimized version of the standard code (100% for the PRS, 98% for the Gilis matrix, see Figs. 4b, 5b). The minimization percentage of most of these codes was lower than that of the standard code (100% for PRS, 85% for the Gilis matrix). Thus, on the fitness landscape, the standard code was found to be closer to its local peak than most of the more robust random codes were from theirs.

**Figure 6:**
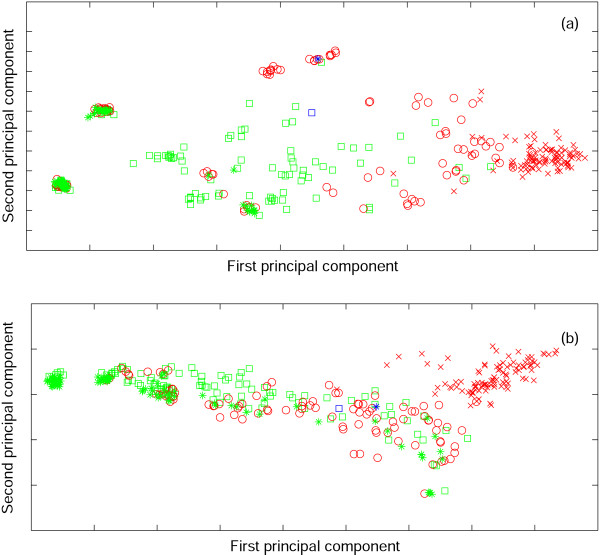
Projection of the code maps onto the plane of the first two principal components (see text for details). Red 'x' signs, random codes, **r**; red circles, codes resulting from optimization of random codes, **o**; green squares, random codes that perform better than the standard code, **R**; green asterisks, codes resulting from optimization of the set **R**, **O**; blue square, the standard code; blue asterisk, the code resulting from the optimization of the standard code. (a) PRS; (b) the Gilis matrix.

To visualize the relationships between the four sets of codes, we employed the following procedure. For each code, we define its map as the vector of the shortest mutational distances between amino acids (there are 190 elements in this vector). The distance between any two codons is calculated as the weighted sum of the number of nucleotide substitutions involved, with the weights assigned according to (2). For instance, in the standard code, Ala is encoded by four codons {GCU, GCC, GCA, GCG}, whereas Arg is encoded by six codons {CGU, CGC, CGA, CGG, AGA, AGG}, and, hence, at least two nucleotide substitutions are required to replace an Arg with an Ala. The distance between these two amino acids, then, is *D*(Arg, Ala) = 1(first position, transition) + 10(second position, transversion) + 0(third position). The code map takes into account these distances without specifying the exact positions of the codons for individual amino acids in the code table. Using principal component analysis (PCA), the code maps for the sets **r**, **R**, **o**, and **O **were projected onto the plane of the first two principal components; the first two principal components account for 20% of the variation in the data in the case of the PRS (Fig. 6a) and for 27% of the variation in the case of the Gilis matrix (Fig. 6b).

The results shown in Fig. 6 indicate that the standard code belongs neither to the set of random codes, **r**, nor to the set **O**, which is the result of minimization of random codes that outperform the natural one. When the distances between the standard code and the codes from the sets **o **and **R **are compared, it is difficult to differentiate between these two sets; however, the code that was obtained as a result of the minimization of the standard code clearly belongs to **o **(the distances from this code to other sets are much greater). Thus, it seems plausible that the standard code belongs in or at least is closest to **o**, i.e., it is a (partially) optimized random code.

**Figure 7:**
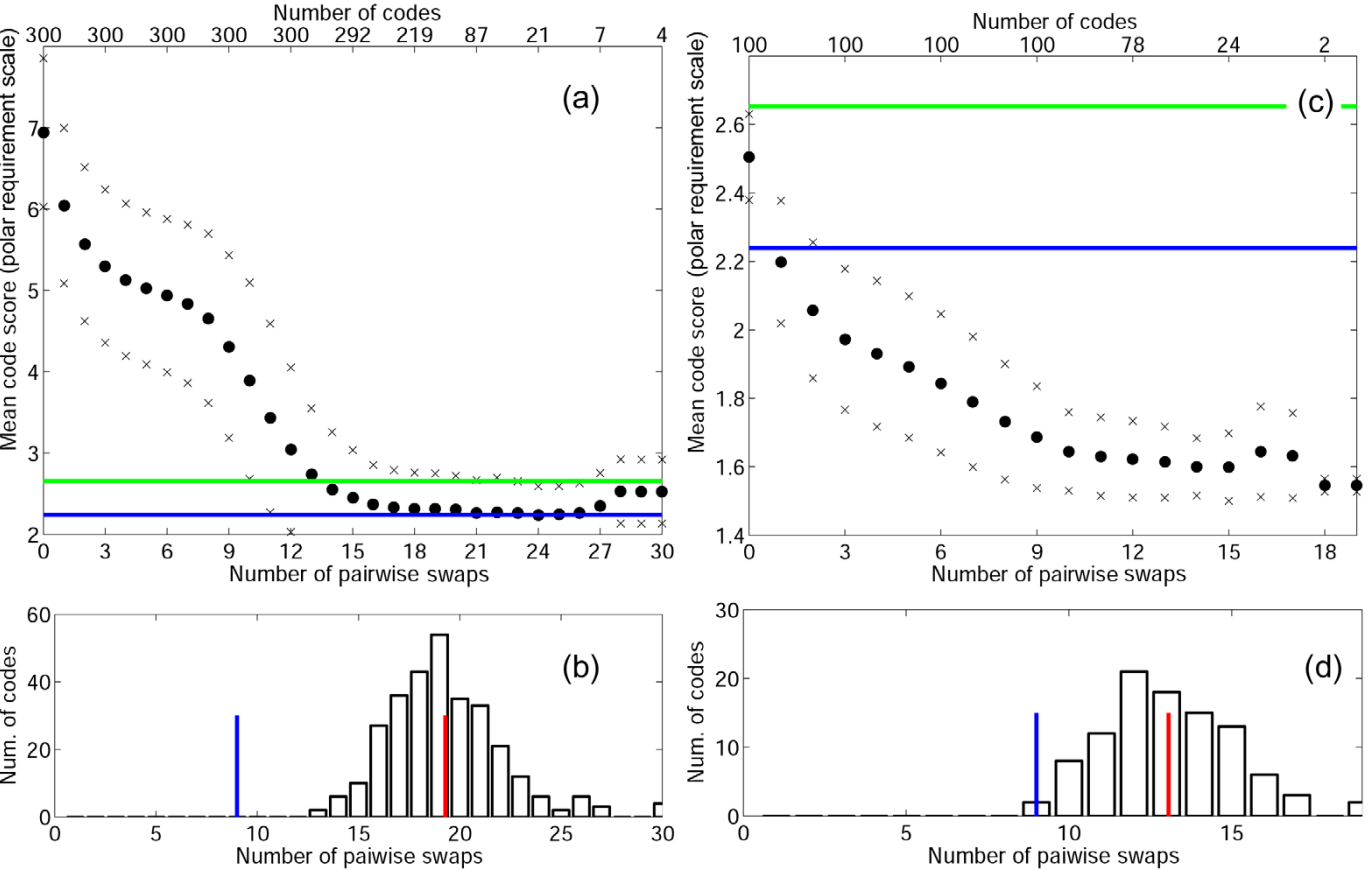
**Evolutionary dynamics of mean code scores in the course of minimization using the PRS as the measure of  amino acid substitution cost**. (a) The black circles show the mean score of the evolving random codes in the course of minimization vs arbitrary time units (pairwise swaps). Crosses show the mean values ± one standard deviation. The green line shows the cost of the standard code, and the blue shows the cost of the code that was obtained by minimization of the standard one. The top x-axis is the number of codes that did not reach their local minimum at the preceding step (starting from 300 random codes). The evolution of each code was followed until the code could not be improved anymore. (b) The number of codes that need exactly *k *pairwise swaps to reach minimum vs *k*; the blue line is the number of steps for the standard code to reach its local fitness peak (9); the red line is the mean of the distribution (19). (c) Same as (a) but the search started with 100 random codes that outperform the standard code. (d) Same as (b) but the search started with 100 random codes that outperform the standard code.

**Figure 8:**
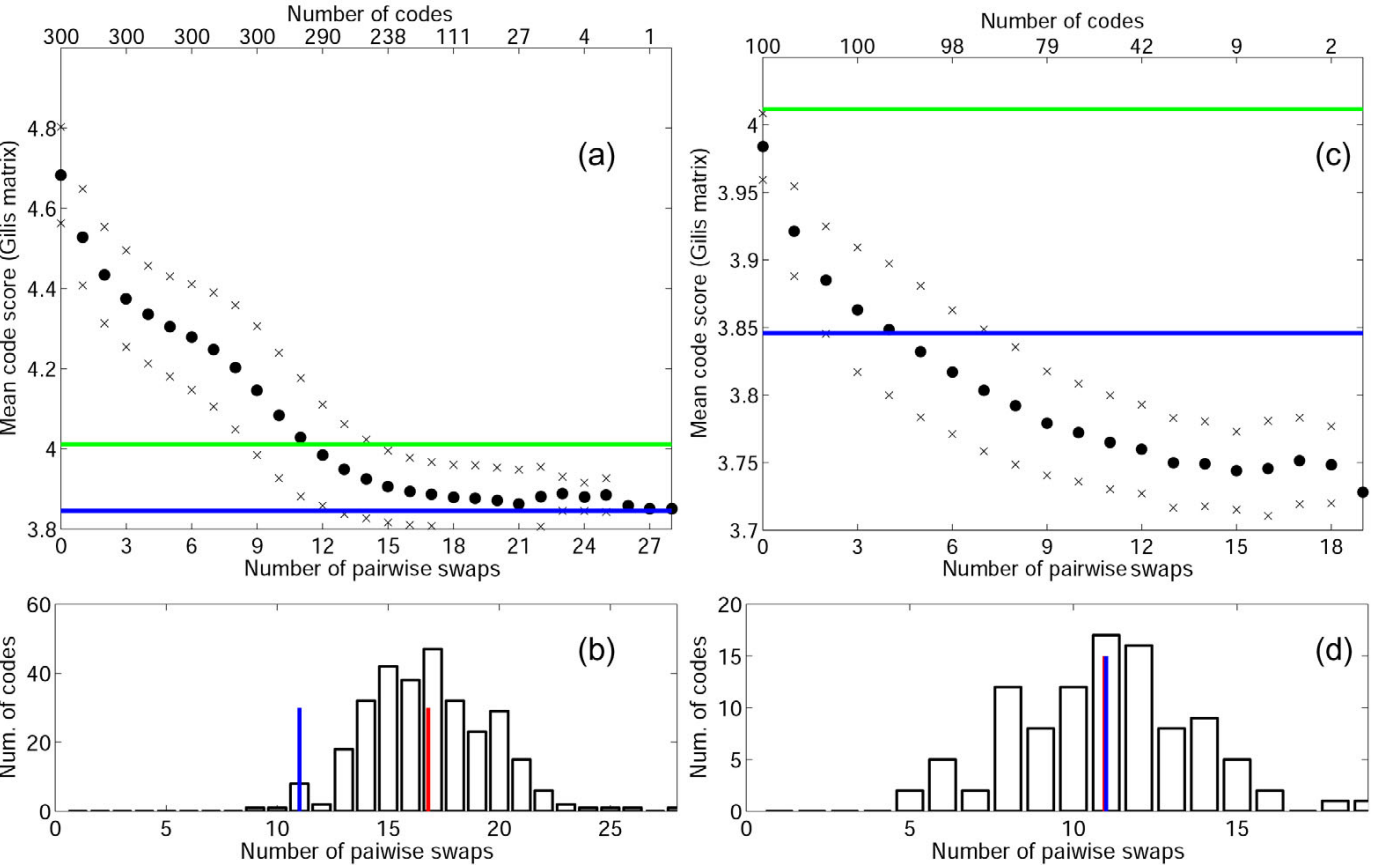
**Evolutionary dynamics of mean code scores in the course of minimization using the Gillis matrix as the measure of amino acid substitution cost**. (a) The black circles show the mean score of the evolving random codes in the course of minimization vs arbitrary time units (pairwise swaps). Crosses show the mean values ± one standard deviation. The green line shows the cost of the standard code, and the blue shows the cost of the code that was obtained by minimization of the standard one. The top x-axis is the number of codes that did not reach their local minimum at the preceding step (starting from 300 random codes). The evolution of each code was followed until the code could not be improved anymore. (b) the number of codes that need exactly *k *pairwise swaps to reach minimum vs *k*; the blue line is the number of steps for the standard code to reach its local fitness peak (9); the red line is the mean of the distribution (19); (c) Same as (a) but the search started with 100 random codes that outperform the standard code; (d) Same as (b) but the search started with 100 random codes that outperform the standard code.

We further examined the length of the evolutionary path to convergence (on a locally optimized code) measured as the number of pairwise swaps that are required to reach the minimum error cost (Figs. 7, 8). For a random code to reach its local minimum, when the PRS is used, 19 swaps are necessary on average (Fig. 7b), and the maximum number of swaps among the 300 tested codes was 30; for the Gilis matrix, the corresponding numbers were 17 and 28 swaps (Fig. 8b). The standard code reached the local minimum after 9 and 11 swaps for the PRS and the Gilis matrix, respectively, which is significantly fewer than the means for the random codes (Figs. 7b, 8b). Notably, on average, the random codes reached, at convergence, the same level of robustness as the standard code although the latter required fewer steps to get there (Figs. 7a, 8a). Put another way, it takes a random code, on average, 8 or 7 swaps (depending on the matrix) to reach the robustness level of the standard code and, then, another 11 or 10 steps to reach the local minimum. Thus, these results supported the notion that the standard genetic code is a partially optimized random code and suggest that it had gone about half of the way along a typical optimization path. We then examined the optimization paths of random codes with a fitness greater than that of the standard code and found that a substantial fraction of these (Figs. 7cd and 8cd) converged at much higher levels of robustness (higher fitness peaks) than the standard code. Again, this result emphasizes the "mediocrity" of the standard code.

## Discussion and Conclusion

In this work, we examined possible evolutionary paths of the genetic code within a restricted domain of the vast parameter space that is, in principle, available for a mapping of 20 amino acids over 64 nucleotide triplets. Specifically, we examined only those codes that possess the same block structure and the same degree of degeneracy as the standard code. It should be noticed, however, that this choice of a small part of the overall, vast code space for further analysis is far from being arbitrary. Indeed, the block structure of the standard code appears to be a direct consequence of the structure of the complex between the cognate tRNA and the codon in mRNA where the third base of the codon plays a minimum role as a specificity determinant. Within this limited – and, presumably, elevated – part of the fitness landscape, we implemented a very simple evolutionary algorithm by taking as an elementary evolutionary step a swap of four-codon or two-codon series. Of course, one has to realize that the model of code's evolution considered here is not necessarily realistic and, technically, should be viewed as a "toy" model. It is conceivable that codon series swaps were not permissible at the stage in the code's evolution when all 20 amino acids have been already recruited. Nevertheless, we believe that the idealized scheme examined here allows for meaningful comparison between the standard code and various classes of random codes.

The evolution of the standard code was compared to the evolution of four sets of codes, namely, purely random codes (**r**), random codes with robustness greater than that of the standard code (**R**), and two sets of codes that resulted from optimization of the first two sets (**o **and **O**, respectively). With the above caveats, the comparison of these sets of codes with the standard code and its locally optimized version yielded several salient observations that held for both measures of amino acid replacements (the PRS and the Gilis matrix) that we employed.

**Figure 9:**
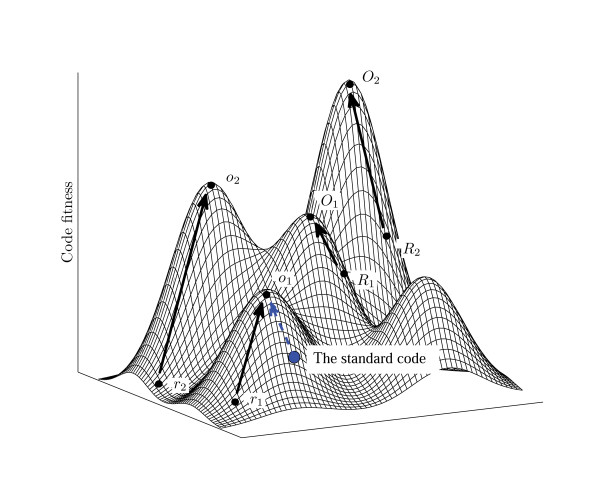
**Evolution of codes in a rugged fitness landscape (a cartoon illustration)**.*r*_1_, *r*_2 _∈ **r**: random codes with the same block structure as the standard code. *o*_1_, *o*_2 _∈ **o**: codes obtained from *r*_1_, *r*_2 _∈ **r**: after optimization. *R*_1_, *R*_2 _∈ **R**: random codes with fitness values greater than the fitness of the standard code. *O*_1_, *O*_2 _∈ **O**: codes obtained from *R*_1_, *R*_2 _∈ **R**: after optimization.

1. The code fitness landscape is extremely rugged such that almost any random initial point (code) tends to its own local optimum (fitness peak).

2. The standard genetic code shows a level of optimization for robustness to errors of translation that can be achieved easily and exceeded by minimization procedure starting from almost any random code.

3. On average, optimization of random codes yielded evolutionary trajectories that converged at the same level of robustness as the optimization path of the standard code; however, the standard code required considerably fewer steps to reach that level than an average random code.

4. When evolutionary trajectories start from random codes whose fitness is comparable to the fitness of the standard code, they typically reach much higher level of optimization than that achieved by optimization of the standard code as an initial condition, and the same holds true for the minimization percentage. Thus, the standard code is much closer to its local minimum (fitness peak) than most of the random codes with similar levels of robustness (Fig. 9).

5. Principal component analysis of the between amino acids distance vectors indicates that the standard code is very different from the sets **r **(all random codes) and **O **(highly optimized codes produced by error cost minimization for random codes that are better than the standard code), and more similar to the codes from **o **(optimized random codes) and **R **(the robust subset of random codes). More importantly, the optimized code produced by minimization of the standard code is much closer to the set of optimized random codes (**o**) than to any other of the analyzed sets of codes.

6. In this fitness landscape, it takes only 15–30 evolutionary steps (codon series swaps) for a typical code to reach the nearest local peak. Notably, the average number of steps that are required for a random code to reach the peak minus the number of steps necessary for the standard code to reach its own peak takes a random code to the same level of robustness as that of the standard code.

Putting all these observations together, we conclude that, in the fitness landscape explored here, the standard genetic code appears to be a point on an evolutionary trajectory from a random point (code) about half the way to the summit of the local peak. Moreover, this peak appears to be rather mediocre, with a huge number of taller peaks existing in the landscape. Of course, it is not known how the code actually evolved but it does seem likely that swapping of codon series was one of the processes involved, at least, at a relatively late stage of code's evolution, when all 20 amino acids have already been recruited. If so, perhaps, the most remarkable thing that we learned, from these modeling exercises, about the standard genetic code is that the null hypothesis on code evolution, namely, that it is a partially optimized random code, could not be rejected. Why did the code's evolution stop where is stopped, i.e., in the middle of the slope of a local fitness peak (Fig. 9), rather than taking it all the way to the summit, especially, as the number of steps required to get there is relatively small? It appears reasonable to view the evolution of the code as a balance of two forces, the positive selection for increasing robustness to errors of translation and the negative selection against any change, i.e., the drive to "freeze an accident". Indeed, codon series swapping is, obviously, a "macromutation" that simultaneously affects all proteins in an organism and would have a deleterious effect that would become increasingly more severe as the complexity of the evolving system increases. This is why, in all likelihood, no such events occurred during advanced stages of life's evolution, i.e., after the cellular organization was established. Conceivably, such an advanced stage in the evolution of life forms was reached before the code reached its local fitness peak, in support of a scenario of code evolution that combines selection for translational robustness with Crick's frozen accident.

## Reviewers' comments

Reviewer 1: David Ardell, The Linnaeus Centre for Bioinformatics, Uppsala University

### General comments

With this manuscript Novozhilov et al. bravely enter the contentious field of modelling the evolution of the genetic code. Novozhilov et al. have contributed some original approaches, concepts and techniques to the field in this work. Although the details of the method are omitted, they convincingly use linear programming to address the question of which amino acid cost matrix would minimize the cost of mistranslation assuming a fixed pattern of translational misreading error. Having solved this problem they then apply their solution to estimate which codon assignments are most deleterious in the standard code. Their conclusion that the placement of arginine is exceedingly maladaptive echoes several earlier uncited works, although their have reached this conclusion by original means. The uncited earlier works include Tolstrup et al. (1994) JMB 243:816, who showed that an artificial neural network trained to learn the standard code segregates amino acids in its internal representation in groups that perfectly correspond to a measure of their hydrophilicity with the exception of arginine. Tolstrup et al. themselves cite earlier work indicating the misfitting assignment of arginine, including Swanson(1984) Bull. Math. Biol. 46:187, Taylor and Coates, and T.H. Jukes (1973) Nature 246:22 who discussed evidence that arginine was a late addition to the genetic code. Finally, arginine is the only amino acid for which strong evidence has been made of a stereochemical association of an amino acid with its codons (Knight and Landweber(2000) RNA 6(4):499).

**Authors' response**: *We appreciate Ardell pointing out this earlier work concerning arginine's position; cited in the revision*.

I also appreciated the application of principal components analysis on representations of genetic codes based on the "codon distances" of amino acids. This is a nice way to measure genetic code similarity in the face of the large equivalence classes of genetic codes under the cost metric that they used (for instance, swapping the two purines or the two pyrimidines in either the first or second codon positions or both, while holding the amino acid assignments fixed, will yield codes with the same cost).

Finally, in terms of incremental improvements to the field, the authors promote an original model for the space of possible genetic codes, and an original mechanism of genetic code change. Their conclusion that the genetic code is a partially optimized random code, is appealing and not controversial to me, although I am quite sure it will continue to be controversial (and perhaps ignored) by others.

However, I take issue with some assumptions and lines of reasoning in this work, which I now outline in decreasing order of relevance to its overall impact:

1. Have the authors meaningfully analyzed a fitness landscape and plausible evolutionary trajectories of genetic codes? This would require adequate measures of 1) fitness and 2) a mechanism of evolutionary change. The authors repeatedly confuse the distinction between "cost" and "fitness" throughout this paper, despite pointing out the distinction themselves at one point in the paper. They also rightly conclude that a true treatment of fitness requires consideration of the population of genes that the genetic code is translating. The important point they neglect is that these genes will also influence how genetic codes can change. Because of this evolutionary constraint that genes place on genetic codes, the likelihood of a swap of amino acids or anticodons between two alloacceptor tRNAs is virtually nil, especially after translation evolved to be accurate enough, so that 20 amino acids could be translated consistently. This precondition is necessary for such a swap (as modelled) to even be meaningful. Perhaps that is why we see no evidence of such radical variation in genetic codes on Earth today. The fact that fitness is not adequately measured in this work and the way that codes change is misrepresented, leaving the basis of their conclusions in doubt. To this I may add that the ruggedness of the cost landscape that they describe is an inevitable consequence, at least in part, of the aforementioned symmetries in the cost metric that they used (leading to equivalence classes of codes as mentioned previously).

On the other hand, the authors' postulated mechanism of swaps of amino acids between pairs of codon blocks is adequate to show that the standard code is sub-optimal, although this has been shown before by others.

**Authors' response**: *There are two distinct points in this comment. One is the alleged confusion between costs and fitness. On this count, we plead not guilty. The cost is defined unequivocally, and the inverse relationship between this cost and fitness is explained right after this definition. In the rest of the text, we speak of reduced cost in the more technical sense and of increased fitness where it comes to a more biologically oriented discussion. We believe this creates clarity rather than confusion*.

*The second point is that the model of code evolution might not be realistic. Here, we plead guilty. The model is deliberately oversimplified to allow straightforward conclusions on the relationships between the standard code and various random codes, and we emphasize this in the revised Discussion*.

2. Is the block structure of the standard genetic code inevitable? There are two components to the block structure: the number of codons assigned to each amino acid and the clustering of redundant codons by the third codon positions. Certainly the "wobble" rules in the third codon position might reasonably be assumed invariant throughout the history of the code. But different tRNA isoacceptors may be altered in their reading capacity through mutations and modifications of their first anticodon bases, i.e. changed in which wobble rule they use. Furthermore, extant altered genetic codes vary in the number of codons assigned to different amino acids. Our own earlier claim (Ardell and Sella, 2001) not withstanding, there is clear evidence in extant life that certain amino acids have most likely inherited or invaded codons from others, thus neither the block structure of the genetic code nor its amino acid expressivity has been invariant throughout its evolution.

**Authors' response**: *In the revised version of the manuscript, we added a caveat emptor (in the discussion section) where we emphasize that we, essentially, explore a toy model of the code's evolution that ignores the expansion of the number of amino acids and involves only codon series swaps. The gist of this paper is the determination of the place of the standard code in the code space, in relation to various classes of random codes, and we believe that, in this respect, the model we employ is adequate*.

3. Using the code itself to decide among different measures of the cost of amino acid replacements, or to infer the nature of translational error, without other evidence, is fallacious. Especially considering the author's own conclusions that the code is non-optimal.

**Authors' response:***We do not actually use the code itself to decide among cost measures. It is another matter that some such measures (e.g., PAM or Blosum matrices) are themselves dependent on the code and therefore hardly appropriate. We do not believe there is anything fallacious in this.*

4. Even though it is widely used, the cited experimental justification for the translational misreading probability scheme in equation 2 is weak, especially in that translational misreading is more transition-biased in misreading of the second codon position than in the first. The data are extremely limited on these points! The cited references are: Friedman and Weinstein, 1966, Woese, 1965 and Parker 1989. In the first reference, only the data translating poly-U are directly interpretable (the poly-UG data has as its highest incorporation Phenylalanine, demonstrative that the mRNA was a random copolymer). Their data (Table 2, page 990) does show a transition-bias of misreading of this one codon as the overall rate of error is increased. But there is no evidence that this bias is greater in the second codon position than the first codon position. In contrast, the data reviewed by Woese (1965) for poly-U show no sign of transition biased misreading in the first codon position at all, but a sign of it in the second codon position. Therefore, the data from these two sources are inconsistent. Furthermore, they arefor only one codon, and Woese writes that the pattern of misreadingof other codons that could be assayed at that time was very different. Importantly, even very recent studies of translational misreading either experimentally in vitro (E. Bouakaz et al. ) or using molecular dynamics simulations (Almlöf et al. (2007) Biochemistry 46:200) center only on the UUU or UUC codons. All authors agree that more studies are necessary with other codons to generalize conclusions. Parker's review of in vivo misreading rates (Table 1, page 277) in no way allow the reader to draw general conclusions regarding the form of translational misreading errors in the different codon positions, other than the general position effect.

On the other hand Kramer and Farabaugh's recent work (cited here in this paper) do demonstrate a greater transition-bias in misreading in position 2 than position 1, in vivo, of all possible one-mutant neighbors to the lysine codon AAA. Nonetheless, this raises the following two questions for me: 1) what translation system under which conditions is the best experimental model for the primordial translation systems under which the genetic code evolved? and 2) Are the highly evolved translation systems studied today biased by actually having co-adapted to the genetic code, so that error frequencies are greatest where costs of errors are weakest?

**Authors' response**: *In the revised manuscript, we are more cautious about the differences in the transition bias between codon positions. The questions asked by Ardell are interesting and relevant. Like he, we currently have no definitive answers*.

As a general point, this paper would benefit very much from separating materials and methods from the results for clarity. In many turns the paper is well written, but in other ways combining M&M and Results makes the paper badly organized and forces the reader to piece together important details of how the work was done from scattered sections of the paper. For example, only incidentally can the reader learn how many different genetic codes were actually analyzed in evaluation of the 4 sets o, r, O and R.

**Authors' response**: *We disagree regarding the amalgamation of M&M and Results. We initially attempted to write the paper in a more traditional manner but found that, in this case, the main methodological approaches were virtually inseparable from the results. The numbers of evaluated codes are now indicated explicitly*.

(p. 9 and elsewhere): Although we (Ardell, 1998, Sella and Ardell,2001, Ardell and Sella 2001, 2002) have shown that 1) mathematical forms such as your eq. 1 are minimized by pairing large terms of p (.|.) with d(.,.), and that 2) codes that imply such pairings are indeed more fit in certain population genetic models, it is only inviting confusions and misunderstanding for the authors' to use the term "fitness" to describe the quantity being optimized. This point is correctly touched on in the paper, but then treated misleadingly elsewhere. May I suggest to call it what it is, which is "cost"?

**Authors' response**: *already addressed above. In general, we do not see conflation of costs and fitness*.

Please detail, regarding software used, how the linear programming problem was solved for reproducibility. Why not provide source code in supplementary methods?

**Authors' response**: *The linear programming problem was solved with a standard routine LPSolve presented in Optimization package of Maple 9.5*.

Reviewer 2: Allan Drummond, Harvard University (nominated by Laura Landweber)

Review of "Evolution of the genetic code: partial optimization of a random code for translational robustness in a rugged fitness landscape", submitted to Biology Direct.

The logical development and main results of the paper are as follows. First, a cost function for genetic codes is specified, and its terms explained (including choices for a distance measure between amino acids). A framework for generating alternative codes is introduced, with a set of assumptions to winnow the search space by roughly 66 orders of magnitude to a tractable set, most importantly the assumption that the block structure of the standard code is a mechanistic consequence of the translational apparatus and therefore non-blocked codes may be safely set aside. The standard code is compared with alternative codes and found to outperform the vast majority of them given a few variants of the assumed cost function. Improvement opportunities for the genetic code are identified by an attempt to minimize the cost function via changes to the distance measure. A greedy minimization algorithm is introduced to search locally for improved variants of an initial code via swaps of codon families. Using this algorithm, the question of whether the standard code should be considered optimized for error minimization is addressed: optimized versions of the standard code and random blocked codes are obtained, and it is found that the standard code's cost, and that of its optimized version, can be matched or beaten by optimized versions of many random blocked codes. The paper's major conclusion is that the standard code is rather unremarkable in its error minimization when compared with other blocked codes.

Overall, I find the subject exciting, the approaches as daring as would be expected from this leading group, and the conclusions interesting. The authors make major assumptions with which I'm not entirely happy, with the justification that they are necessary to make progress. My concern is that unless the assumptions are good, progress is not actually being made, and the topic is better left alone.

I suggest that some assumptions be clarified and buttressed with evidence where they conflict with compelling alternative arguments. The results derived from the greedy minimization algorithm should be substantially revised, as several important claims about this algorithm's output (e.g., that it finds shortest paths) are incorrect. Finally, long-standing questions about the inferences one can draw about evolutionary trajectories, possible or actual, from the output of analytical or computational optimization algorithms should be addressed.

To begin, the simplifying assumptions made to render the search for better codes tractable bear closer examination. In particular, the limitation of searches to codes having the block structure of the universal code is defended, and then used, in a novel way. Given the goal of interpreting simulated trajectories of code modification as informative about the actual process of code evolution (so that, for example, the concept of "close to a fitness peak" and the data in Figures 7 and 8 have meaning in biology as well as the simulation), the authors must establish that the simplifications are biologically reasonable. The burden is heavier here than on other works that make similar assumptions (e.g. the works of Freeland and colleagues) but in which no claims about evolutionary trajectories or the mode of evolutionary exploration are made.

The major assumption leading to the reduction in the search space is that "...the block structure of the code is a direct, mechanistic consequence of the mode of interaction between the ribosome, mRNA, and the cognate tRNA [50]". The premise is worded in a way suggesting that biophysics alone suffice to impose the observed block structure, without invoking selective pressure against mistranslation. This is to my knowledge a completely novel and exciting idea, and substantial evidence should be presented to support it. I was unable to connect the contents of  [50] (Spirin, RNA Biol. 2004) with this premise, and would be helped by exposition on what is being assumed and what is known.

By contrast, the authors might mean the alternative where the block structure of the code arises both from the mode of interaction (e.g., third-position binding contributes most weakly to discrimination, and codon-anticodon mismatches involving transitions are more stable than those involving transversions) and selective pressure for error minimization, which jointly favored a code structure in which third-position transition errors are largely synonymous – a blocked code. That natural selection favors translational error minimization seems obvious; the question at issue is whether the structure of the genetic code contributes alongside other adaptations such as ribosomal structure, kinetic proofreading, synthetase editing activity, biased codon usage for translational accuracy (Akashi Genetics 1994), biased codon usage for error minimization (Archetti JME 2004), tolerance of proteins to mistranslation, etc. If this weaker but plausible assumption is what the authors mean, then it becomes less clear how unblocked codes can be eliminated from consideration in evolutionary pathways, since they are merely assumed to be less fit (as are most codes in the reduced landscape), not unviable, and there are overwhelmingly (~10^66-fold) more of them in the space of all codes, such that selection must work hard to eliminate them.

Indeed, there are extant codes that have a more consistent block structure than the standard genetic code, such as the vertebrate mitochondrial code in which there are no single-codon families (unlike Trp and Met in the standard code). Such block structures are apparently consistent with the mechanism of translation, but are not considered in the present study. I recommend that in the manuscript the authors more muscularly defend the omission of unblocked and differently-blocked codes from evolutionary trajectories.

**Authors' response**: *Indeed, what we mean is that the first two bases contribute substantially more to the recognition of a cognate tRNA than the third, wobble base. This was made explicit in the revision, and the references in support of these differential contributions of bases are cited *([49-51]*). Drummond's point is well taken in that this does not render codes with different block structures impossible "in principle". However, it does make them improbable, and given that for any simulation to run to completion, a relatively small domain of the code space needs to be chosen, fixing the block structure seemed like the best choice. We explain all this in the revision. This may not amount to a "more muscular" defense suggested by Drummond but this is how things are*.

The latter half of the work is mainly concerned with how optimized the standard genetic code is. Given that evolution is a stochastic process, the natural way to think about optimization is to ask what proportion of mutations increase versus decrease the score – a truly optimal code will have zero improvement mutations, and a highly optimized code will be improved by only a tiny fraction of the many possible mutations. Many workers have estimated this proportion by locating the standard code's score relative to a sampled distribution of alternative scores. The authors have taken another approach, using the number of "greedy" codon-family swaps separating a given code from a local optimum to measure how optimized it is. The use of distances between a given code's score and an optimum – here, the minimization percentage, MP – to ascertain the strength of selection has been criticized (Freeland, Knight and Landweber, TiBS 2000), essentially because it improperly treats these distances as a linear measure. The present work compares MP's between codes and is subject to the same criticism. The problem is exacerbated here because the MP is computed relative to each code's local greedy minimum. If, for example, the standard code has an MP of 0.93 and a competing code an MP of 0.8, one cannot conclude that the standard code is more optimized in the sense of having fewer mutations which improve it. That is, the difference between MPs is not equivalent to the difference in optimization level. An alternative is that the codes in the standard code's neighborhood generally score well, so that obtaining a high MP is easy and many mutations would improve the standard code, making it poorly optimized, while the competing code's neighborhood is filled with poor-scoring codes, and it is heavily optimized with few better-scoring codes to move to. Rugged landscapes of the sort explored here are more likely to have such features. The authors should directly address the criticisms regarding MP and search-derived versus stochastically derived measures of optimization, and should sample the local landscape around each code to address the concerns about level of optimization.

**Authors' response**: *In order to compare codes, we employed both a statistical approach and an optimization approach. As a measure of the distance between codes, we used not only the number of codon swaps but also the difference in the error cost values as can be seen in Figs. *3, 4, 5, 7*and *8. *It is not clear why we cannot use MP in the context of the present study. The criticisms of Freeland et al. 2000 addressed the conclusion that, considering the low MP value of the standard code, the code could not evolve under selective pressure to reduce the effect of translation errors. We do not argue with that critique. We use MP only to compare different random codes with the standard code under exactly specified rules for fitness landscape search*.

The mutation-selection balance/non-linear adaptation argument should be considered in the Conclusion where the authors ask, "Why did the code's evolution stop where it stopped?" An answer I glean from much of the error-minimization literature cited in the present work is that it might be wildly improbable for selection to push the level of error-minimization any higher, given countervailing pressure from mutation. If the genetic code is "one in a million" in the sense favored by Freeland and Hurst (JME 1998), that is a high level of optimization by most standards. (A demonstration that many mutations improve that one-in-a-million code would be compelling contrary evidence.) Algorithmic optimization of the sort carried out here is blind to such statistical features – in greedy minimization, the first optimization step is as easy as the last step, because all possible alternatives must be evaluated each time, whereas in a blind sampling-based process such as evolution, the farther uphill one climbs, the more improbable improvement becomes and the less likely it is to persist once attained. This is the essence of Freeland et al. TiBS (2000)'s criticism.

In the same vein, there are several standard evolutionary hypotheses which seem to be missing for why the present genetic code should not be optimal:

- Error minimization was not the sole target of selection. If any other traits were under substantial selection in primordial genomes, and these traits were not perfectly congruent with error minimization, then an evolutionary process favoring increased fitness would yield a sub-optimal genetic code.

- The effective population size was not infinite. Natural selection cannot distinguish fitness differences smaller than the reciprocal of the effective population size. As a consequence, any mutations (to tRNAs, synthetases, release factors, ribosomal components, etc.) which improve the error minimization of the genetic code, but confer a selective advantage below this threshold, would not be expected to reach fixation except by drift. One could in principle estimate how many codes have such a property, and thereby estimate how much optimality would be "left on the table" simply because of the nature of the evolutionary process.

- Mutation-selection balance was achieved. Suppose that error minimization has a bell-shaped distribution, and high levels of error minimization are selectively advantageous, but not infinitely so. The higher error minimization is pushed by selection, the more strongly it is opposed by an increasing proportion of deleterious mutations, until equilibrium – likely at a sub-optimal level of EM – is reached. (Mutation-selection balance is a more mainstream term for the "non-linear adaptation" argument touched on above and briefly by the authors in the Introduction.)

If any of these three standard hypotheses have merit, then the genetic code is expected to be sub-optimal with respect to its robustness to mistranslation. The present work should address these hypotheses in their Discussion, where presently the "balance of two forces" argument addressing the same point is made.

**Authors' response**: *We appreciate these interesting comments. However, in our opinion, this demand puts the plank unrealistically high for any analysis of the evolution of the code. We do not know this "expected non-optimality"*.

The authors measure the stepwise distance from a code to a local peak of the landscape, thereby ascribing significance to this peak. A serious concern is that the algorithm by which this greedy peak is found sheds no light on what general significance the peak possesses. Let us assume that the greedy peak is found to be N steps away from the starting point. The greedy peak is not guaranteed to be a) the closest peak, b) the tallest peak within N steps, or c) the closest or tallest peak approachable using exclusively uphill steps. In a rugged fitness landscape, there is additionally no guarantee that the height of or distance to the greedy peak are informative about the height of or distance to these peaks.

Further, the authors state, "Using this algorithm we can find the shortest evolutionary trajectory from a given starting code to its local minimum of the error cost function (i.e. to a local fitness peak)." This statement is incorrect. Greedy paths will not in general be shortest paths. This can be seen most clearly in Figures 7 and 8, which plot minimization paths of >26 steps, and concluding point #6 which states that a typical code can reach its local peak in 15–30 steps. Given the algorithm used (where a set of mutually accessible codes is uniquely specified by the position of 14 four-codon and 14 two-codon blocks), any code can be changed into any other accessible code in (14-1) + (14-1) = 26 swaps. (The problem is equivalent to a list-reordering problem, and a list of n items can be put in any specified order in n-1 swaps or fewer). It is impossible for a shortest path in the described model to be longer than 26 steps.

As a consequence, Figures 7 and 8 and the accompanying text should be revised. The aim of the experiment is to determine how far various codes are from the local minimum. If "how far" is the shortest-path difference to the closest local minimum, then the data should be retaken using a suitable approach such as dynamic programming. If "how far" is meant in an evolutionary sense, then neither the greedy path nor the shortest path are expected to be representative of evolutionary trajectories, which are blind and therefore subject to entropic constraints as well (cf. the arguments of Freeland and colleagues and the mutation-selection balance comment above). The greedy algorithm is a dubious choice for evolutionary studies, since, for example, the probability of an evolving population moving from one code to the next in any evolutionary model should be a function of the probability of occurrence of the proper mutation and the probability of subsequent fixation, and the greedy algorithm ignores the probability of occurrence altogether. It is easy to imagine deterministic algorithms of equivalent computational complexity which do not ignore such statistics – when all alternatives are being assessed, as in the greedy algorithm, the population mean, median, and so on are deterministic.

**Authors response**: *It is true that, allowing any codon swaps, we can reach any code in (14-1) + (14-1) = 26 steps (not 20). If we knew the final state (the global minimum) this would be an easy problem. Without such knowledge, theoretically, it is possible to find the global minimum using dynamic programming but, practically, this problem is not solvable due to immense number of possible codes. If we allow only swaps that yield the largest fitness increase, then, we find the closest peak (we define a peak as a state from which no codon swaps yield fitness increase) and the tallest peak (because, under the given algorithm only one peak can be reached from any starting point) for this algorithm. We should note that this is, to the best of our understanding, the most reasonable deterministic algorithm of evolution imaginable. It would be a different approach if we added some kind of stochasticity in the landscape search. We decided to use the aforementioned, simple, and therefore, tractable, deterministic, greedy algorithm. In the revised manuscript, we clarified this point in the description of the search algorithm by making it explicit that the algorithm finds an optimization path in which each step involves the maximum possible increase of the code robustness, and added a statement on caveats in the Discussion*.

The validity of a fitness landscape, whether quantitative or illustrative (Fig. 9), derives from that of its metric (distance measure) and fitness function (height). The distance metric chosen here is swaps. That is to say, one step across the landscape equals one exchange in the meaning of two families of codons which encode different amino acids. The authors assert that it is likely the genetic code evolved, at least in part, by such swaps. Woese (BioScience 20(8):471–480 + 485 [1970]) considered several mechanisms for the evolution of the code. Like the authors, he noted that codon reassignments were almost certain to be strongly deleterious, but instead argued that this stumbling block favored an alternate class of evolutionary paths, namely refinement of ancestral stochastic overlapping codon – amino-acid assignments into more precisely delineated families. Evidence addressing Woese's argument, specifically in support the importance of swaps, should be provided early in the manuscript, as the plausibility of the conclusions depend on the acceptance of this premise.

**Authors' response**: *Again, this work is not an attempt to reconstruct a truly realistic scenario for the evolution of the code but rather to determine the status of the standard code in the code space, compared to various sets of random codes, and delineate possible evolutionary links between the standard code and different random codes. This is clarified in the revision*.

The authors note that 9 or 11 swaps is required to take the standard code to its greedy minimum, and refer to this distance as "relatively small"; this interpretation should be justified. As implied above, 9–11 mutations suffice to move any code halfway across the entire vast space of all blocked codes (but recall that the greedy minimum is not necessarily the closest or deepest local minimum). As these swaps are macromutations – several physical mutations would likely be required to swap two four-codon families – the distance would certainly be even larger in reality. I suggest providing the above calculation of the maximum shortest-path length to put whatever distance is found upon revision in perspective.

**Authors' response**: *"Relatively small" means, literally, relatively small with respect to other random codes. It does not seem to us that additional justification is necessary*.

The term "translational robustness" has previously been used to refer to the robustness of individual proteins to mistranslation (Drummond et al. PNAS 2005; Koonin and Wolf, Curr. Op. Biotech. 2006). Here, it is being applied for the first time to the genetic code to denote the idea that certain codes have error spectra which lead to disrupted protein fold or function less than others. These are different phenomena – in the biophysics of how robustness might be obtained and modified, and the scope of consequences if it is altered, among other aspects – and using the same term risks inducing the opposite impression. The phenomenon under consideration has been the subject of many previous works, so the field's common use of "error minimization" might be considered. If a new term is sought, "error robustness" would incorporate the robustness concept while carefully distinguishing it from previous work. If the term must be kept, a short description of how its use here differs from the earlier definition would help to minimize confusion.

**Authors' response**: *We agree, the terminology here deserves more attention. "Translational robustness" could be ambiguous but "error minimization" is not a good phrase either because the structure of the code does not minimize errors, it minimizes their effect. So we went through the manuscript and made changes, speaking of "robustness to translation errors" or, where no ambiguity is perceived, simply, of "robustness"*.

Reviewer 3: Rob Knight, University of Colorado, Boulder

In this manuscript, Novozhilov et al. provide a more detailed exploration of the level of optimality of the genetic code and the evolutionary trajectory of optimization than has previously been available. Specifically, they use a standard approach to measuring the "cost" of a genetic code in terms of the weighted frequency of errors of different severity, and measure the trajectory of codes using a hill-climbing optimization algorithm. They recapture the uncontroversial result that the genetic code is much better at minimizing errors than a random genetic code (as has been shown by many authors), but is at neither a local nor global optimum (as has also been shown previously). However, the results go beyond what has previously been done by comparing the evolutionary trajectory of the standard genetic code to the trajectories of other, random codes to get an estimate of what the overall process should look like.

I believe that the authors overstate their result that the standard genetic code is "not special". Their own results show that it is difficult to explain except as the result of an optimization process: the argument that the standard genetic code is a global optimum is not to my knowledge taken seriously in the field, so the results cannot be seen as overturning it (see discussion between Steve Freeland, Massimo Di Giulio and myself in TiBS in 2000, which is cited appropriately in the paper). Rather, they show that, like most other features of organisms, the genetic code is optimized but not optimal, and probably reflects a range of constraints beyond the specific feature being examined. The manuscript could also benefit from being shortened substantially, as it appears to be relatively long in relation to its news value.

**Authors' response**: *When we say that the standard code is "not special", we mean that it very well could have evolved by partial optimization of a purely random code of the same block structure. Various edits have been made to clarify this but the gist remains. In a way, this is a shift from a half-full glass touted in several previous studies which emphasized that the standard code was "one in a million" or even better than that, to a half-empty glass whereby the standard code appears rather trivial when the entire landscape is considered*.

It is interesting that many of the non-canonical genetic codes in fact do have different block structure or amino acid counts than the canonical genetic codes: indeed, this seems to be the main way that the genetic code is currently evolving (see Knight et al. 2001 Nature Reviews Genetics for a discussion, and Caporaso et al. 2005 J Mol Evol for some simulations involving alternative block structures – the authors might consider citing this latter paper in the discussion of alternative models for code evolution). It might be worth relaxing some of these assumptions in the simulations to model more accurately how we think the code is changing today, although this is perhaps beyond the scope of the present manuscript. We did some work on the optimality of the non-canonical codes in Freeland et al. Mol Biol Evol.

**Authors' response**: *The specifics of the deviant codes in modern organisms, indeed, seem to be beyond the scope of the paper. It is hard to be sure that these in any ways recapitulate the original evolution of the code*.

I think the statement "the standard code is unremarkable" is misleading, for the reasons mentioned above. It is still far better at minimizing errors than the vast majority of codes: perhaps the authors could restate what they mean more clearly.

**Authors' response**: *Restated and qualified in the revision*.

I still disagree that the earlier paper cited, by the same authors, adequately addresses the evidence in favor of a stereochemical effecton the modern code structure. The Caporaso et al. 2005 JME paper shows that there is plenty of "room" for adaptation even if substantial parts of the code were fixed by stereochemistry, for example. Similarly, the present paper does not really discuss the evidence supporting coevolutionary models, although it could be argued that both lines of evidence are outside the scope of this work.

**Authors' response**: *In this context, the previous paper by the same authors (YIW and EVK) is cited as a succinct review of the evidence in support (or lack thereof) of the stereochemical hypothesis. The main conclusion is that there is no compelling evidence in favor of that hypothesis, and we stand by that. As for the being plenty of room for adaptation, we do not seem to be in dispute on this*.

The discussion of the circularity of using PAM matrices should cite Di Giulio's 2001 J Theor Biol paper on the topic, and I also show in my PhD thesis that even very small contamination of a substitution matrix with the genetic code matrix can lead to artifactual statistical significance of the optimality of the genetic code.

**Authors' response**: *The Di Giulio reference was added *[48]*. Unfortunately, at this time, we do not have access to the thesis*.

The assertion that "using this algorithm we can find the shortest evolutionary trajectory from a given starting code to its local minimum of the error cost function" is not true – the shortest trajectory might involve a transition to a worse solution to get to a better one. The algorithm employed can only find the shortest continuously improving path, which might be much longer than a direct route. This point should be clarified in the manuscript.

**Authors' response**: *Indeed, this point has been clarified – see the response to Drummond's comments above*.

## Individual authors' contributions

ASN contributed to the algorithm design, performed the mathematical analysis and wrote the initial draft of the manuscript; YIW contributed to the development of evolutionary models and algorithm design; EVK incepted the study, proposed the evolutionary models, contributed to the algorithm design, and wrote the final version of the manuscript. All authors have read and approved the final version of the manuscript.
